# Supplementary material for: Simultaneous Occurrence of Field Epidemics of Rabbit Hemorrhagic Disease (RHD) in Poland Due to the Co-Presence of Lagovirus europaeus GI.1 (RHDV)/GI.1a (RHDVa) and GI.2 (RHDV2) Genotypes
Source: Viruses. 2025 Sep 26;17(10):1305. doi: 10.3390/v17101305 (PMC12568209; doi:10.3390/v17101305)
Supplement: Supplementary file 1 [file viruses-17-01305-s001.zip › Tab S2 111 seqs VP60 genetic distance (4).pdf]

		1	2	3	4	5	6
1	**WAE_2022_(OR488784)_PL**						
2	**ZWO_2021_(OQ605827)_PL**	0.064					
3	**KOB_2020_(OQ605828)_PL**	0.011	0.058				
4	**LIB_2020_(OQ605829)_PL**	0.182	0.172	0.176			
5	**NRU_2020_(OQ605830)_PL**	0.183	0.175	0.177	0.022		
6	PD_1989_(KP144789)_PL	0.179	0.175	0.172	0.079	0.086	
7	MAL_1994_(KU882093)_RHDV_PL	0.183	0.177	0.178	0.082	0.089	0.020
8	BLA_1994_(KP144792)_PL	0.178	0.171	0.171	0.084	0.088	0.041
9	OPO_2004_(KU882094)_PL	0.178	0.170	0.171	0.086	0.091	0.044
10	GSK_1998_(KU882092)_PL	0.180	0.171	0.171	0.084	0.088	0.042
11	ZD0_2000_(KU882095)_PL	0.178	0.174	0.172	0.090	0.091	0.049
12	GRZ_2004_(KP144791)_PL	0.184	0.173	0.178	0.009	0.020	0.078
13	L14504_2004_(KY679902)_PL	0.185	0.174	0.178	0.009	0.019	0.078
14	W14705_2005_(KY319035)_PL	0.185	0.174	0.178	0.009	0.019	0.078
15	STR_2012_(KF677011)_PL	0.188	0.174	0.181	0.026	0.035	0.080
16	GLE_2013_(KY319032)_PL	0.181	0.171	0.175	0.005	0.024	0.078
17	SKO_2013_(KY319034)_PL	0.187	0.175	0.181	0.025	0.033	0.080
18	RED1_2013_(KY679903)_PL	0.184	0.177	0.181	0.033	0.041	0.074
19	STR2_2013_(KY679904)_PL	0.184	0.171	0.178	0.013	0.023	0.082
20	STR_2014_(KY679905)_PL	0.188	0.175	0.182	0.025	0.032	0.080
21	BIE_2015_(KY319031)_PL	0.187	0.173	0.181	0.023	0.031	0.079
22	F77-3_2015_(MN853658)_PL	0.171	0.158	0.165	0.049	0.055	0.097
23	BBI_2017_(MG602005)_PL	0.180	0.171	0.176	0.016	0.027	0.079
24	RED_2016_(MG602006)_PL	0.050	0.050	0.045	0.178	0.178	0.176
25	VMS_2017_(MG602007)_PL	0.054	0.051	0.048	0.175	0.175	0.173
26	PIN_2018_(MN853660)_PL	0.055	0.058	0.051	0.176	0.177	0.174
27	LIB_2018_(MN853659)_PL	0.012	0.058	0.004	0.176	0.178	0.172
28	WAK_2018_(MN853661)_PL	0.054	0.056	0.049	0.177	0.178	0.172
29	V351_1987_(U54983)_CZ	0.185	0.181	0.178	0.084	0.091	0.016
30	FRG_1989_(M67473)_DE	0.187	0.179	0.179	0.075	0.082	0.012
31	Jena_1993_(EF5585760)_DE	0.179	0.170	0.171	0.085	0.086	0.041
32	Frankfurt5_1996_(EF558573)_DE	0.177	0.171	0.169	0.085	0.089	0.041
33	AST89_1989_(Z49271)_ES	0.185	0.175	0.176	0.083	0.086	0.040
34	RHDV-SD_1989_(Z29514)_FR	0.186	0.178	0.177	0.085	0.089	0.046
35	95-10_1995_(MT628287)_FR	0.181	0.171	0.175	0.087	0.092	0.044
36	00-21_2000_(MH190418)_FR	0.178	0.174	0.171	0.086	0.088	0.046
37	09-02_2009_(MT628289)_FR	0.179	0.171	0.171	0.081	0.083	0.035
38	09-03_2009_(MT628290)_FR	0.178	0.174	0.174	0.095	0.096	0.063
39	96VLT000113_1995_(MT819374)_S	0.176	0.171	0.169	0.085	0.088	0.047
40	12VLT000099_2012_(MT819377)_S	0.179	0.168	0.173	0.102	0.099	0.070
41	BS89_1989_(X87607)_IT	0.177	0.168	0.168	0.084	0.086	0.034
42	CB137_1995_(JX886002)_PT	0.186	0.182	0.177	0.101	0.101	0.064
43	CB156_1997_(JF438967)_PT	0.184	0.177	0.175	0.096	0.099	0.058
44	CB194_2006_(JX886001)_PT	0.188	0.176	0.181	0.105	0.104	0.081
45	Woodcroft_2005_(KT006741)_AUS	0.184	0.179	0.177	0.090	0.095	0.037
46	Triptis_1996_(EF558583)_DE	0.181	0.171	0.176	0.030	0.037	0.065
47	Erfurt_1996_(EF558581)_DE	0.182	0.172	0.177	0.013	0.022	0.078
48	Rossi_2002_(EF558584)_DE	0.181	0.172	0.175	0.037	0.043	0.070
49	P175_1999_(KY622129)_PT	0.182	0.175	0.178	0.026	0.034	0.072
50	RHDV-Hokkaido_2002_(AB300693)_JPN	0.188	0.180	0.182	0.037	0.039	0.077
51	WHNRH_2005_(DQ280493)_CN	0.186	0.171	0.179	0.018	0.024	0.084
52	RHDV_2014_(MK895974)_CN	0.186	0.177	0.181	0.030	0.037	0.082
53	WIN-AH-2011-OTH-026_(KY235676)_CAN	0.184	0.170	0.177	0.033	0.039	0.084
54	Iowa_2000_(AF258618)_USA	0.181	0.171	0.176	0.028	0.034	0.073
55	IN-05_2005_(EU003578)_USA	0.182	0.172	0.176	0.020	0.026	0.083

56	13-165_2013_(MN737112)_FR	0.050	0.046	0.045	0.172	0.174	0.174
57	16-35OOd_2016_(MN738377)_FR	0.019	0.055	0.013	0.173	0.174	0.172
58	NL-2016_(MN061492)_NL	0.052	0.054	0.047	0.176	0.177	0.174
59	N11_2011_(KM87868)_ES	0.049	0.041	0.041	0.172	0.174	0.175
60	Zar11-11_2010_(KP129398)_ES	0.048	0.045	0.040	0.174	0.176	0.174
61	CBAnd1_2012_(KP090976)_ES	0.052	0.046	0.044	0.174	0.175	0.174
62	Seg08-12_2012_(KP129396)_ES	0.046	0.043	0.041	0.174	0.174	0.175
63	Rij06-12_2012_(KP129395)_ES	0.052	0.047	0.047	0.175	0.177	0.177
64	16PLM1_2016_(MF407653)_ES	0.056	0.050	0.050	0.174	0.175	0.176
65	CBVal16_2012_(KM979445)_PT	0.047	0.043	0.041	0.177	0.178	0.178
66	Algarve1_2013_(KF442961)_PT	0.047	0.046	0.041	0.176	0.177	0.175
67	7-13_Barrancos_2013_(KF442963)_PT	0.047	0.045	0.040	0.175	0.176	0.174
68	10A-13_Barrancos_2013_(KF442964)_PT	0.046	0.044	0.040	0.176	0.177	0.175
69	CBAlgarve14-1_2014_(KM115714)_PT	0.051	0.048	0.044	0.178	0.179	0.179
70	CBAlgarve14-3_2014_(KM115715)_PT	0.050	0.047	0.044	0.178	0.178	0.179
71	CBEstremoz_14-1_2014_(KM115681)_PT	0.051	0.049	0.043	0.177	0.177	0.175
72	CBMert_14-1_2014_(KM115712)_PT	0.050	0.047	0.043	0.177	0.178	0.178
73	CBMert14-2_2014_(KM115713)_PT	0.050	0.047	0.042	0.178	0.179	0.179
74	SOS089_2014_(MG763936)_PT	0.052	0.050	0.046	0.178	0.177	0.174
75	SOS158_2015_(MG763947)_PT	0.053	0.051	0.046	0.176	0.177	0.172
76	PSM2_2016_(MF407654)_PT	0.051	0.046	0.046	0.178	0.179	0.175
77	CBPico17-1_2017_(MF407651)_PT	0.054	0.054	0.048	0.181	0.181	0.177
78	CBPico-17-2_2017_(MF407652)_PT	0.054	0.054	0.048	0.181	0.181	0.177
79	CBMad17-1_2017_(MF407655)_PT	0.057	0.057	0.050	0.174	0.174	0.171
80	CBMad17-2_2017_(MF407656)_PT	0.053	0.048	0.047	0.178	0.180	0.176
81	CBMad17-3_2017_(MF407657)_PT	0.051	0.047	0.046	0.179	0.181	0.177
82	RHDV/GER-NW/D51-1.L00911_2014_(LR899189)_DE	0.047	0.044	0.041	0.178	0.178	0.178
83	EI327.L03607/2016_(LR899157)_DE	0.050	0.053	0.045	0.174	0.175	0.172
84	BLMT-1_2015_(KT280060)_AUS	0.052	0.049	0.046	0.175	0.176	0.179
85	AZ1_2020_(MT506237)_USA	0.040	0.056	0.032	0.176	0.177	0.173
86	NY1_2020_(MT506236)_USA	0.039	0.060	0.034	0.175	0.174	0.175
87	NY2_2020_(MT506235)_USA	0.039	0.060	0.034	0.175	0.174	0.175
88	WIN-AH-2016-OTH-0018_(KY235675)_CAN	0.052	0.047	0.046	0.173	0.174	0.171
89	WIN-AH-2019-OTH-0032_(MT900574)_CAN	0.037	0.054	0.031	0.171	0.171	0.172
90	Senasica20_2020_(OM973948)_MX	0.041	0.055	0.033	0.175	0.176	0.172
91	RHDV2-S25_2019_(MW789242)_GH	0.061	0.062	0.056	0.177	0.178	0.171
92	Touza_1_2019_(MZ913394)_TN	0.062	0.042	0.056	0.168	0.172	0.171
93	Ibaraki-1_2019_(LC749423)_JPN	0.056	0.053	0.051	0.175	0.175	0.174
94	Chiba-1_2020_(LC749425)_JPN	0.058	0.054	0.053	0.174	0.175	0.173
95	06-11_2006_(MN737115)_FR	0.184	0.182	0.182	0.162	0.163	0.148
96	08-133_2008_(MN746289)_FR	0.184	0.185	0.183	0.161	0.162	0.150
97	09-48_2009_(MN737116)_FR	0.184	0.184	0.181	0.160	0.159	0.144
98	MRCV_2001_(GQ166866)_USA	0.188	0.187	0.184	0.166	0.173	0.159
99	RCV-A1_MIC-07_2007_(EU871528)_AUS	0.208	0.197	0.203	0.201	0.203	0.202
100	Australia_1_2007_(KX357690)_AUS	0.205	0.196	0.198	0.192	0.195	0.188
101	AUS/NSW/ANN-1/2014/04_(KY628306)_AUS	0.188	0.184	0.181	0.056	0.061	0.094
102	Otago/NZ-37/2018_(OM372665)_NZ	0.055	0.055	0.047	0.167	0.167	0.167
103	10-28_2010_(MN737113)_FR	0.046	0.047	0.039	0.171	0.172	0.171
104	10-32_2010_(MN737114)_FR	0.041	0.046	0.034	0.169	0.170	0.171
105	K5_08Q712_2008_(MF598301)_AUS	0.185	0.177	0.179	0.041	0.047	0.077
106	ACT/AIN-5_2017_(MW460019)_AUS	0.054	0.050	0.049	0.179	0.179	0.177
107	NSW/CAR-3/2016_(MF598302)_AUS	0.056	0.049	0.049	0.181	0.180	0.179
108	EBHSV-GD_(Z69620)_FR	0.298	0.286	0.298	0.293	0.296	0.294
109	Ca11_2011_(KC345614)_IT	0.175	0.169	0.169	0.096	0.099	0.067
110	NanBu_2011_(JQ815391)_CN	0.189	0.178	0.183	0.050	0.053	0.072
111	RCV_1996_(X96868)_IT	0.185	0.178	0.184	0.145	0.143	0.137

7	8	9	10	11	12	13	14	15	16	17	18	19	20	21	22
0.043															
0.045	0.007														
0.041	0.031	0.033													
0.053	0.038	0.041	0.023												
0.081	0.083	0.085	0.083	0.089											
0.081	0.082	0.085	0.082	0.089	0.003										
0.081	0.082	0.085	0.082	0.089	0.002	0.002									
0.083	0.086	0.088	0.086	0.092	0.023	0.022	0.023								
0.081	0.083	0.085	0.083	0.091	0.011	0.011	0.011	0.028							
0.083	0.086	0.088	0.086	0.092	0.022	0.020	0.021	0.007	0.026						
0.077	0.080	0.082	0.082	0.085	0.029	0.028	0.028	0.034	0.034	0.033					
0.084	0.083	0.085	0.084	0.091	0.011	0.010	0.010	0.027	0.015	0.025	0.034				
0.083	0.085	0.088	0.085	0.092	0.021	0.019	0.020	0.008	0.026	0.004	0.032	0.025			
0.082	0.085	0.088	0.085	0.092	0.020	0.018	0.019	0.006	0.025	0.003	0.031	0.023	0.002		
0.098	0.097	0.099	0.099	0.106	0.045	0.046	0.046	0.049	0.048	0.047	0.054	0.051	0.046	0.045	
0.083	0.085	0.086	0.085	0.091	0.011	0.010	0.010	0.029	0.018	0.029	0.033	0.018	0.028	0.027	0.051
0.178	0.174	0.174	0.177	0.176	0.179	0.179	0.179	0.178	0.177	0.178	0.177	0.178	0.179	0.178	0.164
0.174	0.165	0.169	0.171	0.171	0.176	0.175	0.176	0.177	0.174	0.177	0.174	0.174	0.177	0.176	0.162
0.174	0.168	0.168	0.171	0.174	0.177	0.177	0.177	0.177	0.175	0.178	0.175	0.177	0.178	0.177	0.163
0.177	0.171	0.171	0.172	0.173	0.178	0.178	0.178	0.181	0.175	0.181	0.181	0.178	0.182	0.181	0.165
0.172	0.168	0.168	0.171	0.174	0.178	0.178	0.178	0.179	0.176	0.179	0.177	0.177	0.179	0.178	0.164
0.023	0.046	0.049	0.046	0.057	0.083	0.082	0.082	0.085	0.083	0.084	0.082	0.086	0.084	0.083	0.100
0.018	0.040	0.043	0.040	0.050	0.075	0.074	0.074	0.078	0.075	0.078	0.075	0.078	0.078	0.077	0.094
0.043	0.029	0.032	0.015	0.016	0.084	0.083	0.083	0.088	0.084	0.086	0.082	0.085	0.086	0.086	0.101
0.043	0.003	0.008	0.030	0.037	0.085	0.084	0.084	0.086	0.085	0.086	0.081	0.085	0.086	0.086	0.098
0.047	0.046	0.050	0.045	0.055	0.081	0.081	0.081	0.079	0.082	0.082	0.076	0.084	0.082	0.081	0.098
0.053	0.050	0.054	0.050	0.060	0.084	0.084	0.084	0.084	0.085	0.085	0.078	0.084	0.085	0.084	0.101
0.048	0.023	0.025	0.034	0.040	0.086	0.086	0.086	0.088	0.086	0.086	0.082	0.088	0.086	0.086	0.101
0.048	0.038	0.041	0.021	0.027	0.086	0.085	0.085	0.089	0.085	0.089	0.085	0.087	0.088	0.088	0.104
0.040	0.023	0.027	0.026	0.031	0.078	0.078	0.078	0.081	0.080	0.081	0.077	0.081	0.080	0.080	0.096
0.061	0.057	0.059	0.046	0.053	0.096	0.095	0.095	0.099	0.095	0.100	0.100	0.096	0.098	0.098	0.116
0.050	0.035	0.039	0.022	0.009	0.086	0.086	0.086	0.091	0.086	0.091	0.083	0.089	0.090	0.090	0.101
0.068	0.058	0.060	0.046	0.055	0.101	0.102	0.102	0.105	0.101	0.106	0.101	0.102	0.106	0.106	0.117
0.041	0.020	0.024	0.026	0.032	0.081	0.081	0.081	0.083	0.084	0.083	0.078	0.084	0.082	0.082	0.097
0.068	0.068	0.071	0.069	0.077	0.099	0.099	0.099	0.097	0.101	0.099	0.096	0.102	0.099	0.099	0.115
0.064	0.061	0.065	0.061	0.066	0.094	0.094	0.094	0.093	0.095	0.094	0.091	0.096	0.094	0.094	0.108
0.081	0.081	0.085	0.079	0.080	0.103	0.103	0.103	0.104	0.105	0.103	0.101	0.106	0.103	0.102	0.117
0.044	0.056	0.059	0.055	0.062	0.089	0.088	0.088	0.090	0.089	0.090	0.084	0.091	0.090	0.089	0.099
0.068	0.071	0.074	0.072	0.077	0.026	0.025	0.025	0.033	0.032	0.031	0.020	0.031	0.030	0.029	0.054
0.079	0.082	0.085	0.081	0.089	0.010	0.009	0.009	0.019	0.015	0.018	0.025	0.013	0.017	0.016	0.042
0.072	0.074	0.076	0.076	0.081	0.033	0.032	0.032	0.040	0.039	0.038	0.027	0.038	0.037	0.036	0.057
0.074	0.078	0.081	0.079	0.084	0.022	0.021	0.021	0.026	0.027	0.026	0.016	0.027	0.025	0.024	0.050
0.079	0.083	0.086	0.084	0.089	0.033	0.033	0.033	0.036	0.039	0.034	0.030	0.037	0.034	0.033	0.059
0.086	0.088	0.091	0.087	0.095	0.015	0.014	0.014	0.030	0.020	0.028	0.037	0.018	0.027	0.026	0.051
0.086	0.085	0.088	0.086	0.091	0.026	0.026	0.027	0.030	0.033	0.028	0.037	0.032	0.027	0.026	0.032
0.087	0.088	0.090	0.089	0.095	0.029	0.029	0.029	0.033	0.034	0.032	0.040	0.034	0.032	0.031	0.048
0.075	0.078	0.080	0.077	0.084	0.024	0.022	0.023	0.032	0.030	0.030	0.022	0.028	0.030	0.029	0.052
0.084	0.086	0.090	0.086	0.092	0.016	0.017	0.017	0.032	0.022	0.030	0.036	0.021	0.029	0.028	0.051

0.177	0.172	0.174	0.173	0.175	0.173	0.174	0.174	0.175	0.171	0.176	0.173	0.172	0.176	0.175	0.159
0.177	0.171	0.172	0.171	0.170	0.175	0.175	0.175	0.177	0.172	0.177	0.175	0.175	0.178	0.177	0.162
0.174	0.168	0.168	0.171	0.173	0.177	0.177	0.177	0.178	0.175	0.178	0.175	0.177	0.178	0.177	0.164
0.177	0.172	0.172	0.172	0.173	0.173	0.174	0.174	0.175	0.171	0.176	0.173	0.172	0.176	0.175	0.158
0.177	0.171	0.171	0.174	0.176	0.175	0.175	0.175	0.176	0.173	0.177	0.174	0.174	0.177	0.175	0.161
0.177	0.172	0.174	0.172	0.174	0.175	0.175	0.175	0.177	0.173	0.178	0.174	0.174	0.178	0.177	0.160
0.178	0.174	0.173	0.174	0.174	0.174	0.175	0.175	0.177	0.172	0.177	0.175	0.174	0.177	0.176	0.158
0.178	0.173	0.174	0.175	0.177	0.177	0.176	0.176	0.178	0.174	0.178	0.177	0.175	0.178	0.177	0.161
0.177	0.173	0.173	0.173	0.175	0.174	0.175	0.175	0.175	0.171	0.176	0.174	0.175	0.175	0.175	0.160
0.179	0.174	0.175	0.175	0.177	0.177	0.178	0.178	0.179	0.175	0.180	0.177	0.177	0.180	0.179	0.161
0.178	0.174	0.174	0.177	0.175	0.177	0.177	0.177	0.179	0.175	0.179	0.177	0.176	0.179	0.178	0.161
0.177	0.174	0.174	0.176	0.175	0.176	0.177	0.177	0.177	0.174	0.178	0.176	0.175	0.178	0.177	0.160
0.178	0.174	0.174	0.177	0.175	0.177	0.177	0.177	0.178	0.175	0.178	0.177	0.176	0.178	0.177	0.160
0.182	0.177	0.176	0.180	0.179	0.179	0.179	0.179	0.181	0.177	0.182	0.179	0.178	0.182	0.181	0.163
0.182	0.177	0.176	0.179	0.179	0.178	0.179	0.179	0.181	0.177	0.182	0.178	0.178	0.182	0.181	0.162
0.179	0.175	0.177	0.177	0.176	0.177	0.178	0.178	0.179	0.175	0.180	0.178	0.177	0.180	0.179	0.161
0.181	0.177	0.175	0.179	0.179	0.178	0.178	0.178	0.180	0.176	0.182	0.178	0.177	0.182	0.181	0.161
0.182	0.178	0.177	0.179	0.180	0.179	0.179	0.179	0.181	0.177	0.183	0.179	0.178	0.183	0.182	0.162
0.177	0.172	0.172	0.175	0.176	0.178	0.179	0.179	0.178	0.177	0.180	0.177	0.178	0.180	0.179	0.164
0.175	0.170	0.170	0.173	0.174	0.177	0.177	0.177	0.177	0.175	0.178	0.175	0.176	0.178	0.177	0.162
0.178	0.175	0.176	0.177	0.177	0.178	0.179	0.179	0.179	0.177	0.180	0.176	0.178	0.180	0.179	0.164
0.180	0.174	0.174	0.177	0.177	0.181	0.182	0.182	0.181	0.179	0.183	0.179	0.181	0.183	0.182	0.168
0.180	0.174	0.174	0.177	0.177	0.181	0.182	0.182	0.181	0.179	0.183	0.179	0.181	0.183	0.182	0.168
0.175	0.174	0.175	0.172	0.169	0.175	0.175	0.175	0.178	0.173	0.179	0.176	0.174	0.179	0.178	0.162
0.179	0.175	0.177	0.177	0.179	0.179	0.179	0.179	0.180	0.177	0.181	0.177	0.178	0.181	0.179	0.165
0.179	0.176	0.177	0.177	0.178	0.179	0.180	0.180	0.181	0.178	0.181	0.177	0.179	0.181	0.180	0.165
0.179	0.175	0.175	0.179	0.179	0.178	0.179	0.179	0.179	0.177	0.180	0.178	0.178	0.180	0.179	0.162
0.172	0.168	0.168	0.170	0.172	0.175	0.175	0.175	0.176	0.173	0.177	0.174	0.175	0.177	0.175	0.162
0.181	0.175	0.175	0.177	0.181	0.175	0.176	0.176	0.180	0.174	0.181	0.177	0.176	0.181	0.179	0.161
0.179	0.170	0.171	0.172	0.170	0.178	0.178	0.178	0.179	0.175	0.179	0.178	0.177	0.181	0.179	0.162
0.182	0.175	0.177	0.177	0.175	0.177	0.177	0.177	0.180	0.176	0.179	0.177	0.176	0.179	0.178	0.165
0.182	0.175	0.177	0.177	0.175	0.177	0.177	0.177	0.180	0.176	0.179	0.177	0.176	0.179	0.178	0.165
0.175	0.169	0.169	0.171	0.172	0.172	0.173	0.173	0.175	0.171	0.175	0.175	0.172	0.175	0.174	0.160
0.178	0.170	0.170	0.171	0.171	0.172	0.173	0.173	0.176	0.172	0.175	0.176	0.172	0.175	0.174	0.160
0.179	0.169	0.171	0.171	0.169	0.177	0.178	0.178	0.178	0.174	0.179	0.177	0.177	0.180	0.179	0.162
0.170	0.169	0.169	0.168	0.171	0.177	0.177	0.177	0.177	0.176	0.178	0.175	0.177	0.178	0.177	0.165
0.177	0.175	0.176	0.171	0.171	0.169	0.169	0.169	0.170	0.167	0.169	0.172	0.168	0.169	0.167	0.161
0.175	0.169	0.169	0.171	0.173	0.175	0.176	0.176	0.176	0.175	0.177	0.175	0.176	0.177	0.175	0.162
0.174	0.169	0.169	0.170	0.172	0.175	0.175	0.175	0.175	0.174	0.176	0.174	0.175	0.176	0.175	0.162
0.153	0.151	0.155	0.155	0.156	0.164	0.164	0.164	0.167	0.164	0.166	0.160	0.167	0.165	0.163	0.172
0.154	0.150	0.154	0.155	0.150	0.161	0.162	0.162	0.166	0.163	0.165	0.156	0.166	0.163	0.162	0.169
0.148	0.146	0.150	0.149	0.150	0.160	0.160	0.160	0.161	0.161	0.160	0.153	0.164	0.159	0.157	0.165
0.157	0.155	0.161	0.154	0.154	0.169	0.169	0.169	0.168	0.170	0.168	0.162	0.170	0.169	0.168	0.175
0.206	0.203	0.203	0.202	0.199	0.205	0.204	0.204	0.203	0.202	0.202	0.200	0.204	0.203	0.203	0.211
0.192	0.186	0.188	0.184	0.186	0.194	0.196	0.196	0.198	0.190	0.196	0.190	0.198	0.197	0.196	0.200
0.094	0.098	0.102	0.099	0.103	0.055	0.054	0.054	0.058	0.057	0.056	0.051	0.058	0.057	0.056	0.077
0.171	0.171	0.172	0.171	0.172	0.168	0.168	0.168	0.169	0.166	0.169	0.168	0.167	0.170	0.169	0.156
0.174	0.167	0.169	0.168	0.171	0.171	0.172	0.172	0.172	0.169	0.173	0.172	0.171	0.174	0.173	0.157
0.174	0.169	0.170	0.169	0.171	0.170	0.171	0.171	0.171	0.168	0.172	0.171	0.169	0.173	0.172	0.156
0.077	0.088	0.090	0.088	0.094	0.038	0.037	0.037	0.043	0.041	0.041	0.039	0.042	0.041	0.040	0.064
0.179	0.179	0.180	0.180	0.181	0.180	0.181	0.181	0.185	0.178	0.185	0.180	0.181	0.185	0.184	0.167
0.181	0.179	0.179	0.181	0.182	0.181	0.182	0.182	0.186	0.179	0.186	0.181	0.182	0.186	0.185	0.165
0.292	0.290	0.291	0.290	0.297	0.295	0.295	0.295	0.299	0.292	0.299	0.293	0.294	0.297	0.297	0.300
0.065	0.061	0.063	0.044	0.053	0.096	0.096	0.096	0.100	0.096	0.100	0.096	0.096	0.098	0.098	0.112
0.071	0.075	0.078	0.074	0.079	0.046	0.046	0.046	0.049	0.051	0.046	0.043	0.050	0.047	0.046	0.068
0.139	0.140	0.143	0.138	0.134	0.143	0.143	0.143	0.148	0.145	0.149	0.143	0.143	0.147	0.146	0.156

23	24	25	26	27	28	29	30	31	32	33	34	35	36	37	38
0.175															
0.174	0.032														
0.176	0.039	0.043													
0.176	0.046	0.047	0.050												
0.177	0.038	0.040	0.013	0.048											
0.086	0.182	0.179	0.179	0.177	0.178										
0.079	0.182	0.178	0.179	0.178	0.178	0.011									
0.084	0.174	0.168	0.171	0.171	0.169	0.047	0.040								
0.086	0.172	0.165	0.167	0.169	0.166	0.046	0.040	0.028							
0.082	0.179	0.178	0.180	0.178	0.180	0.047	0.041	0.048	0.046						
0.085	0.179	0.178	0.182	0.179	0.180	0.053	0.047	0.053	0.050	0.008					
0.088	0.174	0.168	0.171	0.175	0.171	0.049	0.041	0.029	0.022	0.049	0.054				
0.086	0.178	0.174	0.176	0.171	0.174	0.051	0.045	0.020	0.037	0.054	0.058	0.039			
0.081	0.172	0.169	0.170	0.172	0.171	0.040	0.034	0.023	0.023	0.038	0.043	0.025	0.031		
0.096	0.177	0.176	0.171	0.173	0.172	0.067	0.063	0.044	0.056	0.072	0.077	0.060	0.050	0.050	
0.087	0.174	0.168	0.172	0.170	0.172	0.054	0.047	0.015	0.034	0.053	0.057	0.037	0.026	0.029	0.053
0.102	0.174	0.172	0.172	0.171	0.169	0.075	0.070	0.046	0.057	0.074	0.078	0.060	0.052	0.051	0.037
0.083	0.171	0.165	0.167	0.168	0.168	0.040	0.034	0.022	0.020	0.040	0.044	0.022	0.031	0.013	0.051
0.100	0.180	0.181	0.181	0.179	0.182	0.067	0.061	0.074	0.067	0.032	0.036	0.071	0.076	0.063	0.091
0.095	0.177	0.178	0.180	0.177	0.180	0.065	0.057	0.063	0.061	0.025	0.030	0.063	0.067	0.054	0.084
0.106	0.180	0.178	0.182	0.183	0.182	0.085	0.079	0.081	0.082	0.052	0.057	0.086	0.085	0.078	0.101
0.091	0.181	0.178	0.180	0.175	0.179	0.033	0.034	0.057	0.056	0.058	0.064	0.061	0.059	0.051	0.077
0.029	0.175	0.172	0.175	0.176	0.177	0.071	0.064	0.071	0.071	0.068	0.071	0.074	0.073	0.065	0.091
0.017	0.177	0.174	0.177	0.177	0.177	0.082	0.075	0.082	0.083	0.079	0.082	0.084	0.083	0.077	0.095
0.036	0.174	0.172	0.175	0.175	0.175	0.074	0.068	0.076	0.074	0.070	0.072	0.077	0.078	0.072	0.095
0.027	0.177	0.174	0.177	0.178	0.179	0.077	0.070	0.079	0.078	0.075	0.078	0.080	0.082	0.072	0.095
0.039	0.181	0.179	0.181	0.182	0.184	0.082	0.075	0.084	0.084	0.077	0.081	0.085	0.085	0.078	0.098
0.022	0.178	0.174	0.178	0.179	0.178	0.088	0.080	0.088	0.088	0.086	0.091	0.091	0.089	0.083	0.099
0.033	0.182	0.180	0.180	0.181	0.180	0.085	0.079	0.088	0.086	0.082	0.085	0.089	0.088	0.082	0.101
0.036	0.174	0.172	0.172	0.177	0.175	0.089	0.082	0.091	0.088	0.086	0.089	0.091	0.092	0.086	0.105
0.029	0.175	0.174	0.176	0.176	0.177	0.078	0.071	0.077	0.078	0.074	0.077	0.081	0.081	0.072	0.095
0.023	0.179	0.174	0.175	0.176	0.175	0.086	0.079	0.086	0.088	0.085	0.086	0.088	0.088	0.082	0.097

0.171	0.023	0.022	0.035	0.044	0.034	0.179	0.179	0.171	0.171	0.179	0.180	0.170	0.174	0.170	0.173
0.173	0.038	0.041	0.044	0.013	0.040	0.177	0.178	0.168	0.169	0.175	0.177	0.174	0.171	0.169	0.174
0.176	0.035	0.038	0.009	0.046	0.009	0.179	0.179	0.169	0.167	0.180	0.181	0.169	0.174	0.169	0.171
0.171	0.018	0.022	0.032	0.040	0.030	0.178	0.179	0.170	0.170	0.179	0.180	0.171	0.175	0.171	0.175
0.173	0.021	0.024	0.034	0.040	0.033	0.180	0.180	0.172	0.169	0.179	0.181	0.171	0.176	0.172	0.178
0.172	0.024	0.025	0.036	0.044	0.035	0.180	0.180	0.171	0.171	0.179	0.181	0.171	0.174	0.172	0.176
0.172	0.018	0.023	0.033	0.041	0.032	0.182	0.182	0.172	0.174	0.180	0.181	0.174	0.177	0.174	0.177
0.175	0.025	0.026	0.037	0.046	0.034	0.181	0.181	0.173	0.171	0.181	0.182	0.172	0.177	0.173	0.178
0.174	0.029	0.032	0.032	0.050	0.032	0.179	0.181	0.171	0.171	0.181	0.182	0.169	0.174	0.170	0.174
0.175	0.019	0.022	0.032	0.041	0.031	0.183	0.184	0.173	0.172	0.183	0.184	0.172	0.177	0.174	0.177
0.175	0.017	0.025	0.034	0.041	0.033	0.182	0.182	0.175	0.172	0.181	0.182	0.174	0.179	0.174	0.178
0.174	0.016	0.024	0.033	0.041	0.032	0.181	0.181	0.174	0.172	0.181	0.182	0.173	0.179	0.173	0.177
0.175	0.016	0.023	0.033	0.040	0.032	0.182	0.182	0.175	0.172	0.181	0.182	0.174	0.179	0.174	0.178
0.176	0.019	0.028	0.038	0.044	0.037	0.185	0.185	0.178	0.175	0.182	0.185	0.175	0.182	0.177	0.181
0.175	0.019	0.027	0.037	0.043	0.036	0.186	0.185	0.178	0.175	0.182	0.184	0.175	0.182	0.177	0.181
0.175	0.022	0.028	0.039	0.045	0.037	0.182	0.182	0.175	0.173	0.180	0.181	0.175	0.180	0.174	0.181
0.175	0.018	0.027	0.037	0.043	0.036	0.185	0.184	0.177	0.175	0.181	0.184	0.175	0.182	0.176	0.180
0.176	0.018	0.027	0.037	0.043	0.036	0.186	0.185	0.178	0.176	0.181	0.184	0.176	0.183	0.177	0.181
0.177	0.021	0.029	0.039	0.046	0.037	0.181	0.181	0.173	0.170	0.176	0.176	0.172	0.178	0.173	0.177
0.175	0.022	0.030	0.039	0.047	0.037	0.179	0.179	0.171	0.168	0.175	0.175	0.170	0.175	0.171	0.177
0.177	0.019	0.029	0.036	0.045	0.036	0.182	0.182	0.175	0.173	0.181	0.183	0.173	0.179	0.174	0.176
0.179	0.023	0.034	0.043	0.050	0.042	0.184	0.184	0.175	0.172	0.181	0.182	0.175	0.178	0.172	0.180
0.179	0.023	0.034	0.043	0.050	0.042	0.184	0.184	0.175	0.172	0.181	0.182	0.175	0.178	0.172	0.180
0.173	0.034	0.037	0.048	0.051	0.049	0.179	0.179	0.170	0.172	0.178	0.179	0.172	0.174	0.169	0.177
0.177	0.021	0.031	0.038	0.047	0.038	0.183	0.183	0.176	0.174	0.182	0.185	0.174	0.179	0.175	0.177
0.178	0.020	0.030	0.037	0.046	0.037	0.184	0.184	0.175	0.174	0.182	0.184	0.174	0.180	0.175	0.176
0.178	0.015	0.024	0.032	0.042	0.031	0.184	0.184	0.178	0.173	0.184	0.185	0.175	0.181	0.174	0.178
0.174	0.034	0.036	0.009	0.044	0.008	0.178	0.178	0.168	0.166	0.178	0.179	0.168	0.174	0.168	0.171
0.174	0.023	0.027	0.036	0.045	0.034	0.184	0.184	0.175	0.174	0.182	0.184	0.175	0.180	0.176	0.179
0.176	0.043	0.047	0.049	0.033	0.049	0.180	0.181	0.169	0.168	0.178	0.179	0.173	0.170	0.169	0.173
0.175	0.044	0.046	0.047	0.033	0.047	0.184	0.184	0.174	0.174	0.181	0.182	0.176	0.175	0.175	0.178
0.175	0.044	0.046	0.047	0.033	0.047	0.184	0.184	0.174	0.174	0.181	0.182	0.176	0.175	0.175	0.178
0.168	0.034	0.038	0.043	0.045	0.041	0.178	0.177	0.169	0.168	0.176	0.177	0.169	0.172	0.169	0.172
0.171	0.040	0.041	0.040	0.030	0.041	0.179	0.180	0.170	0.168	0.178	0.181	0.172	0.172	0.169	0.175
0.175	0.041	0.046	0.047	0.034	0.047	0.179	0.181	0.169	0.168	0.178	0.178	0.172	0.172	0.169	0.172
0.176	0.044	0.047	0.019	0.055	0.018	0.175	0.175	0.166	0.167	0.176	0.178	0.168	0.171	0.166	0.168
0.167	0.044	0.044	0.053	0.055	0.052	0.178	0.177	0.170	0.173	0.174	0.178	0.175	0.172	0.171	0.174
0.175	0.041	0.041	0.017	0.050	0.017	0.179	0.179	0.169	0.168	0.178	0.180	0.171	0.175	0.170	0.172
0.174	0.043	0.043	0.019	0.052	0.018	0.179	0.179	0.168	0.167	0.177	0.181	0.169	0.174	0.169	0.173
0.164	0.175	0.175	0.177	0.182	0.177	0.154	0.152	0.154	0.150	0.153	0.154	0.151	0.154	0.150	0.158
0.162	0.176	0.179	0.181	0.183	0.181	0.155	0.153	0.151	0.149	0.152	0.153	0.152	0.154	0.148	0.157
0.160	0.170	0.176	0.178	0.181	0.177	0.149	0.147	0.147	0.143	0.148	0.150	0.147	0.149	0.146	0.158
0.171	0.185	0.178	0.184	0.184	0.181	0.161	0.158	0.155	0.155	0.161	0.165	0.158	0.156	0.155	0.157
0.199	0.206	0.203	0.208	0.204	0.208	0.205	0.202	0.200	0.203	0.201	0.203	0.200	0.200	0.198	0.205
0.196	0.196	0.197	0.196	0.201	0.197	0.192	0.189	0.186	0.185	0.193	0.196	0.188	0.186	0.181	0.186
0.058	0.181	0.179	0.184	0.182	0.185	0.100	0.095	0.098	0.099	0.094	0.098	0.102	0.100	0.098	0.111
0.167	0.045	0.044	0.049	0.049	0.046	0.172	0.172	0.169	0.169	0.174	0.174	0.171	0.171	0.167	0.170
0.169	0.032	0.035	0.041	0.038	0.041	0.178	0.176	0.167	0.165	0.175	0.177	0.168	0.172	0.169	0.172
0.168	0.030	0.033	0.038	0.034	0.037	0.174	0.175	0.168	0.168	0.172	0.174	0.170	0.172	0.165	0.172
0.044	0.180	0.176	0.179	0.179	0.180	0.081	0.074	0.089	0.089	0.084	0.087	0.091	0.092	0.086	0.102
0.178	0.025	0.031	0.037	0.049	0.034	0.184	0.184	0.178	0.177	0.184	0.185	0.177	0.182	0.178	0.178
0.178	0.024	0.030	0.039	0.050	0.036	0.186	0.186	0.179	0.178	0.184	0.186	0.179	0.183	0.179	0.180
0.294	0.294	0.290	0.296	0.297	0.292	0.296	0.291	0.294	0.291	0.288	0.289	0.290	0.294	0.289	0.299
0.096	0.172	0.169	0.171	0.168	0.168	0.072	0.066	0.044	0.060	0.071	0.074	0.058	0.047	0.053	0.034
0.050	0.181	0.181	0.184	0.183	0.185	0.077	0.070	0.072	0.075	0.072	0.076	0.077	0.076	0.071	0.091
0.144	0.180	0.174	0.179	0.184	0.178	0.141	0.137	0.137	0.139	0.140	0.142	0.139	0.139	0.136	0.139

39	40	41	42	43	44	45	46	47	48	49	50	51	52	53	54
0.054															
0.030	0.053														
0.075	0.094	0.063													
0.065	0.086	0.055	0.027												
0.079	0.099	0.081	0.054	0.050											
0.060	0.080	0.052	0.078	0.073	0.092										
0.074	0.094	0.068	0.087	0.082	0.094	0.077									
0.085	0.102	0.079	0.099	0.094	0.105	0.086	0.025								
0.078	0.099	0.073	0.089	0.084	0.094	0.078	0.011	0.032							
0.082	0.099	0.075	0.094	0.089	0.101	0.080	0.015	0.019	0.022						
0.086	0.100	0.080	0.094	0.088	0.102	0.084	0.029	0.031	0.034	0.020					
0.091	0.102	0.085	0.103	0.098	0.106	0.096	0.034	0.018	0.041	0.029	0.040				
0.088	0.105	0.082	0.100	0.094	0.102	0.085	0.032	0.023	0.037	0.032	0.041	0.035			
0.092	0.108	0.085	0.101	0.097	0.107	0.092	0.036	0.028	0.042	0.034	0.043	0.035	0.034		
0.081	0.099	0.075	0.090	0.084	0.093	0.080	0.019	0.024	0.026	0.016	0.027	0.031	0.034	0.038	
0.089	0.101	0.084	0.103	0.098	0.108	0.094	0.034	0.018	0.039	0.030	0.039	0.023	0.033	0.037	0.033

0.172	0.169	0.166	0.182	0.178	0.177	0.179	0.171	0.172	0.172	0.174	0.179	0.171	0.179	0.170	0.172
0.167	0.171	0.168	0.177	0.175	0.179	0.175	0.173	0.174	0.172	0.174	0.180	0.177	0.178	0.174	0.174
0.171	0.169	0.166	0.182	0.179	0.182	0.180	0.175	0.175	0.174	0.177	0.182	0.177	0.180	0.172	0.176
0.171	0.169	0.168	0.182	0.179	0.176	0.179	0.171	0.172	0.171	0.174	0.180	0.171	0.178	0.170	0.172
0.174	0.171	0.168	0.184	0.181	0.179	0.179	0.172	0.174	0.171	0.174	0.181	0.173	0.179	0.171	0.173
0.171	0.169	0.168	0.181	0.178	0.177	0.179	0.172	0.172	0.171	0.174	0.181	0.172	0.179	0.171	0.174
0.172	0.173	0.171	0.184	0.180	0.176	0.183	0.172	0.174	0.172	0.175	0.179	0.174	0.178	0.172	0.174
0.175	0.172	0.169	0.184	0.182	0.180	0.181	0.175	0.174	0.175	0.176	0.181	0.174	0.181	0.174	0.176
0.173	0.171	0.169	0.182	0.181	0.178	0.178	0.172	0.173	0.174	0.174	0.179	0.174	0.178	0.170	0.172
0.174	0.172	0.170	0.185	0.182	0.180	0.184	0.175	0.176	0.176	0.178	0.183	0.175	0.182	0.174	0.177
0.174	0.174	0.171	0.183	0.179	0.179	0.183	0.174	0.177	0.173	0.176	0.181	0.175	0.180	0.175	0.175
0.174	0.174	0.170	0.184	0.180	0.179	0.184	0.173	0.176	0.172	0.175	0.180	0.174	0.179	0.173	0.175
0.174	0.174	0.171	0.183	0.179	0.179	0.183	0.174	0.177	0.173	0.176	0.181	0.175	0.180	0.174	0.175
0.177	0.178	0.174	0.184	0.180	0.181	0.186	0.177	0.179	0.177	0.179	0.184	0.177	0.184	0.175	0.177
0.177	0.177	0.174	0.183	0.179	0.180	0.186	0.177	0.178	0.176	0.180	0.184	0.177	0.183	0.174	0.177
0.175	0.177	0.171	0.182	0.179	0.178	0.184	0.174	0.177	0.174	0.177	0.181	0.175	0.179	0.175	0.175
0.177	0.177	0.173	0.182	0.179	0.179	0.185	0.176	0.178	0.175	0.178	0.183	0.176	0.182	0.174	0.176
0.178	0.178	0.174	0.182	0.179	0.179	0.186	0.177	0.179	0.177	0.179	0.184	0.177	0.184	0.175	0.177
0.174	0.172	0.169	0.181	0.177	0.181	0.181	0.174	0.177	0.174	0.177	0.180	0.175	0.183	0.173	0.177
0.172	0.172	0.167	0.180	0.177	0.181	0.179	0.174	0.175	0.173	0.176	0.179	0.174	0.181	0.171	0.175
0.175	0.175	0.170	0.183	0.179	0.179	0.181	0.174	0.177	0.174	0.177	0.181	0.178	0.182	0.173	0.177
0.174	0.175	0.169	0.184	0.180	0.181	0.182	0.177	0.180	0.178	0.178	0.183	0.179	0.185	0.176	0.178
0.174	0.175	0.169	0.184	0.180	0.181	0.182	0.177	0.180	0.178	0.178	0.183	0.179	0.185	0.176	0.178
0.168	0.171	0.167	0.181	0.177	0.177	0.179	0.173	0.175	0.175	0.175	0.179	0.174	0.179	0.174	0.174
0.177	0.175	0.171	0.185	0.181	0.179	0.182	0.175	0.178	0.175	0.177	0.182	0.178	0.182	0.174	0.177
0.176	0.175	0.171	0.184	0.181	0.180	0.182	0.175	0.178	0.175	0.178	0.182	0.179	0.183	0.174	0.177
0.177	0.177	0.171	0.184	0.184	0.181	0.184	0.177	0.177	0.176	0.178	0.181	0.177	0.182	0.173	0.178
0.170	0.169	0.165	0.181	0.178	0.180	0.178	0.173	0.174	0.172	0.175	0.181	0.177	0.178	0.172	0.174
0.176	0.175	0.172	0.185	0.182	0.181	0.185	0.175	0.175	0.174	0.177	0.181	0.172	0.180	0.171	0.176
0.168	0.172	0.167	0.180	0.177	0.181	0.180	0.175	0.177	0.177	0.177	0.183	0.178	0.179	0.175	0.176
0.173	0.173	0.172	0.184	0.181	0.179	0.181	0.175	0.175	0.174	0.177	0.183	0.176	0.179	0.175	0.176
0.173	0.173	0.172	0.184	0.181	0.179	0.181	0.175	0.175	0.174	0.177	0.183	0.176	0.179	0.175	0.176
0.169	0.169	0.164	0.179	0.177	0.177	0.178	0.171	0.171	0.173	0.174	0.179	0.172	0.179	0.171	0.172
0.169	0.172	0.168	0.181	0.178	0.177	0.178	0.172	0.171	0.171	0.174	0.180	0.174	0.175	0.172	0.173
0.167	0.171	0.166	0.181	0.176	0.182	0.179	0.175	0.176	0.176	0.177	0.182	0.178	0.179	0.174	0.175
0.170	0.169	0.165	0.179	0.175	0.179	0.176	0.174	0.175	0.174	0.177	0.182	0.177	0.179	0.173	0.175
0.169	0.172	0.167	0.178	0.177	0.174	0.177	0.167	0.167	0.169	0.171	0.175	0.168	0.175	0.166	0.169
0.171	0.169	0.168	0.181	0.179	0.178	0.181	0.173	0.175	0.172	0.175	0.181	0.177	0.178	0.172	0.174
0.170	0.170	0.168	0.182	0.178	0.178	0.181	0.172	0.174	0.172	0.175	0.180	0.177	0.178	0.171	0.174
0.152	0.155	0.148	0.165	0.157	0.168	0.158	0.157	0.164	0.162	0.161	0.164	0.164	0.165	0.164	0.161
0.147	0.151	0.147	0.164	0.157	0.168	0.157	0.154	0.162	0.157	0.156	0.161	0.162	0.161	0.161	0.158
0.146	0.154	0.144	0.161	0.155	0.163	0.151	0.152	0.159	0.157	0.155	0.155	0.160	0.158	0.158	0.154
0.153	0.161	0.154	0.170	0.164	0.169	0.158	0.162	0.168	0.162	0.163	0.164	0.167	0.169	0.168	0.161
0.200	0.198	0.196	0.200	0.198	0.207	0.206	0.203	0.205	0.204	0.201	0.204	0.205	0.207	0.202	0.203
0.186	0.181	0.185	0.193	0.190	0.195	0.197	0.191	0.196	0.194	0.192	0.192	0.199	0.196	0.195	0.191
0.099	0.110	0.099	0.105	0.106	0.110	0.100	0.050	0.053	0.054	0.041	0.037	0.061	0.061	0.058	0.049
0.170	0.168	0.165	0.177	0.173	0.181	0.172	0.165	0.167	0.166	0.169	0.174	0.169	0.171	0.165	0.168
0.168	0.168	0.164	0.178	0.175	0.175	0.179	0.169	0.170	0.172	0.170	0.178	0.169	0.177	0.168	0.170
0.169	0.170	0.165	0.173	0.171	0.174	0.177	0.167	0.169	0.168	0.168	0.176	0.171	0.175	0.167	0.168
0.092	0.106	0.086	0.099	0.098	0.105	0.085	0.037	0.037	0.041	0.032	0.041	0.045	0.044	0.047	0.039
0.178	0.177	0.175	0.186	0.183	0.183	0.185	0.178	0.180	0.178	0.180	0.184	0.177	0.186	0.175	0.181
0.180	0.177	0.176	0.184	0.182	0.182	0.188	0.179	0.182	0.180	0.182	0.184	0.178	0.187	0.177	0.182
0.295	0.295	0.289	0.290	0.290	0.298	0.296	0.293	0.298	0.290	0.292	0.293	0.295	0.303	0.299	0.292
0.053	0.036	0.052	0.089	0.084	0.103	0.079	0.088	0.093	0.094	0.094	0.098	0.099	0.099	0.102	0.094
0.077	0.091	0.072	0.086	0.085	0.094	0.081	0.033	0.045	0.040	0.036	0.041	0.054	0.051	0.057	0.041
0.135	0.140	0.135	0.151	0.146	0.152	0.143	0.140	0.145	0.143	0.141	0.140	0.146	0.146	0.148	0.145

55 56 57 58 59 60 61 62 63 64 65 66 67 68 69 70

0.172															
0.173	0.038														
0.175	0.031	0.040													
0.171	0.013	0.033	0.027												
0.173	0.016	0.033	0.030	0.008											
0.173	0.016	0.037	0.032	0.008	0.011										
0.172	0.014	0.034	0.029	0.008	0.013	0.014									
0.174	0.012	0.040	0.033	0.013	0.016	0.018	0.016								
0.171	0.025	0.042	0.029	0.021	0.022	0.026	0.023	0.027							
0.174	0.009	0.034	0.027	0.009	0.012	0.013	0.009	0.011	0.020						
0.175	0.015	0.036	0.030	0.011	0.013	0.015	0.008	0.017	0.023	0.010					
0.174	0.015	0.036	0.029	0.010	0.012	0.015	0.008	0.016	0.023	0.009	0.002				
0.175	0.014	0.035	0.029	0.009	0.012	0.014	0.007	0.016	0.022	0.009	0.001	0.001			
0.178	0.021	0.037	0.034	0.016	0.019	0.020	0.015	0.023	0.029	0.016	0.014	0.013	0.013		
0.178	0.020	0.037	0.033	0.016	0.019	0.020	0.015	0.022	0.028	0.015	0.013	0.013	0.012	0.003	
0.175	0.020	0.040	0.034	0.015	0.017	0.020	0.013	0.020	0.028	0.015	0.006	0.006	0.006	0.019	0.018
0.177	0.020	0.036	0.033	0.015	0.018	0.020	0.014	0.022	0.027	0.015	0.013	0.012	0.012	0.002	0.002
0.178	0.020	0.036	0.033	0.015	0.018	0.020	0.014	0.022	0.027	0.015	0.013	0.012	0.012	0.002	0.002
0.175	0.022	0.040	0.034	0.018	0.018	0.020	0.018	0.024	0.030	0.018	0.016	0.016	0.015	0.019	0.018
0.175	0.023	0.040	0.035	0.018	0.019	0.020	0.018	0.025	0.030	0.019	0.017	0.016	0.016	0.019	0.019
0.178	0.022	0.040	0.032	0.019	0.020	0.022	0.018	0.024	0.027	0.018	0.016	0.016	0.015	0.016	0.016
0.181	0.027	0.043	0.038	0.022	0.023	0.026	0.021	0.029	0.032	0.020	0.020	0.019	0.019	0.023	0.023
0.181	0.027	0.043	0.038	0.022	0.023	0.026	0.021	0.029	0.032	0.020	0.020	0.019	0.019	0.023	0.023
0.175	0.027	0.047	0.044	0.027	0.029	0.029	0.025	0.032	0.037	0.026	0.020	0.020	0.019	0.032	0.032
0.178	0.024	0.041	0.034	0.020	0.022	0.024	0.019	0.026	0.026	0.020	0.018	0.018	0.017	0.018	0.018
0.179	0.023	0.041	0.033	0.020	0.022	0.023	0.019	0.025	0.028	0.019	0.018	0.017	0.016	0.018	0.017
0.177	0.015	0.036	0.029	0.011	0.014	0.016	0.010	0.016	0.023	0.011	0.010	0.009	0.009	0.013	0.013
0.173	0.029	0.038	0.004	0.026	0.029	0.030	0.027	0.031	0.027	0.026	0.028	0.027	0.027	0.032	0.032
0.175	0.020	0.040	0.031	0.018	0.020	0.022	0.017	0.024	0.029	0.017	0.016	0.016	0.015	0.019	0.018
0.177	0.043	0.027	0.046	0.041	0.043	0.043	0.041	0.046	0.049	0.040	0.042	0.041	0.041	0.046	0.045
0.172	0.042	0.028	0.044	0.037	0.039	0.041	0.037	0.042	0.047	0.038	0.040	0.039	0.039	0.043	0.043
0.172	0.042	0.028	0.044	0.037	0.039	0.041	0.037	0.042	0.047	0.038	0.040	0.039	0.039	0.043	0.043
0.171	0.030	0.040	0.039	0.026	0.027	0.029	0.029	0.032	0.035	0.027	0.030	0.029	0.029	0.032	0.033
0.171	0.038	0.024	0.037	0.033	0.035	0.037	0.033	0.039	0.043	0.034	0.036	0.035	0.034	0.039	0.039
0.177	0.042	0.029	0.044	0.040	0.041	0.042	0.040	0.044	0.048	0.039	0.041	0.040	0.040	0.044	0.044
0.175	0.040	0.046	0.013	0.035	0.039	0.041	0.037	0.041	0.036	0.037	0.039	0.038	0.037	0.041	0.041
0.167	0.038	0.050	0.049	0.036	0.036	0.037	0.036	0.040	0.043	0.036	0.039	0.039	0.038	0.041	0.042
0.175	0.036	0.044	0.013	0.032	0.035	0.037	0.033	0.037	0.033	0.033	0.034	0.034	0.033	0.039	0.038
0.174	0.037	0.047	0.015	0.034	0.037	0.039	0.035	0.039	0.034	0.034	0.036	0.036	0.035	0.040	0.040
0.163	0.172	0.180	0.177	0.176	0.179	0.174	0.175	0.176	0.177	0.176	0.175	0.176	0.175	0.179	0.178
0.161	0.175	0.180	0.181	0.177	0.178	0.176	0.177	0.179	0.180	0.179	0.177	0.178	0.177	0.181	0.180
0.160	0.174	0.177	0.177	0.174	0.178	0.174	0.175	0.178	0.176	0.178	0.176	0.177	0.176	0.178	0.178
0.168	0.184	0.182	0.185	0.184	0.185	0.182	0.186	0.186	0.186	0.186	0.188	0.186	0.187	0.187	0.188
0.206	0.204	0.203	0.207	0.203	0.206	0.205	0.206	0.202	0.205	0.205	0.207	0.207	0.206	0.205	0.204
0.196	0.192	0.196	0.197	0.192	0.194	0.195	0.193	0.193	0.193	0.194	0.194	0.193	0.194	0.193	0.192
0.061	0.179	0.181	0.186	0.182	0.181	0.181	0.181	0.183	0.180	0.184	0.182	0.181	0.182	0.184	0.185
0.166	0.042	0.040	0.043	0.034	0.039	0.040	0.040	0.044	0.049	0.040	0.042	0.041	0.041	0.045	0.045
0.169	0.028	0.032	0.038	0.021	0.024	0.026	0.027	0.029	0.038	0.024	0.028	0.027	0.027	0.032	0.031
0.168	0.029	0.026	0.034	0.019	0.023	0.025	0.025	0.029	0.035	0.025	0.027	0.027	0.026	0.032	0.032
0.044	0.178	0.177	0.179	0.177	0.177	0.175	0.178	0.179	0.179	0.181	0.179	0.178	0.179	0.182	0.183
0.178	0.024	0.044	0.032	0.022	0.023	0.025	0.020	0.026	0.028	0.021	0.019	0.019	0.018	0.023	0.022
0.181	0.026	0.044	0.033	0.022	0.024	0.025	0.019	0.027	0.029	0.022	0.019	0.018	0.018	0.021	0.020
0.289	0.292	0.292	0.293	0.292	0.294	0.294	0.293	0.293	0.296	0.292	0.296	0.296	0.295	0.294	0.294
0.096	0.168	0.167	0.167	0.168	0.169	0.168	0.171	0.171	0.169	0.171	0.172	0.172	0.172	0.176	0.175
0.054	0.179	0.184	0.184	0.181	0.181	0.180	0.181	0.182	0.180	0.184	0.182	0.181	0.182	0.185	0.185
0.141	0.174	0.182	0.181	0.176	0.178	0.175	0.176	0.178	0.177	0.179	0.177	0.178	0.177	0.178	0.179

71	72	73	74	75	76	77	78	79	80	81	82	83	84	85	86

0.018															
0.016	0.001														
0.021	0.018	0.018													
0.022	0.018	0.018	0.003												
0.021	0.015	0.015	0.022	0.023											
0.025	0.022	0.022	0.025	0.025	0.021										
0.025	0.022	0.022	0.025	0.025	0.021	0.000									
0.025	0.031	0.031	0.032	0.033	0.033	0.036	0.036								
0.023	0.017	0.017	0.024	0.025	0.002	0.023	0.023	0.034							
0.022	0.016	0.016	0.023	0.024	0.001	0.022	0.022	0.034	0.003						
0.015	0.012	0.012	0.016	0.016	0.016	0.019	0.019	0.027	0.018	0.017					
0.033	0.031	0.031	0.033	0.033	0.032	0.036	0.036	0.043	0.033	0.033	0.027				
0.021	0.018	0.018	0.022	0.023	0.022	0.026	0.026	0.034	0.024	0.023	0.015	0.029			
0.044	0.044	0.044	0.047	0.047	0.043	0.047	0.047	0.051	0.045	0.044	0.041	0.045	0.046		
0.042	0.042	0.042	0.044	0.045	0.041	0.047	0.047	0.053	0.043	0.042	0.039	0.043	0.044	0.034	
0.042	0.042	0.042	0.044	0.045	0.041	0.047	0.047	0.053	0.043	0.042	0.039	0.043	0.044	0.034	0.000
0.033	0.032	0.032	0.036	0.036	0.033	0.036	0.036	0.043	0.035	0.034	0.029	0.037	0.032	0.046	0.044
0.039	0.038	0.038	0.040	0.041	0.039	0.043	0.043	0.046	0.041	0.040	0.035	0.037	0.040	0.028	0.012
0.043	0.043	0.043	0.046	0.046	0.042	0.046	0.046	0.050	0.044	0.043	0.040	0.044	0.045	0.004	0.034
0.043	0.040	0.040	0.042	0.043	0.042	0.048	0.048	0.053	0.044	0.043	0.038	0.013	0.040	0.053	0.053
0.043	0.041	0.041	0.044	0.044	0.039	0.045	0.045	0.049	0.040	0.040	0.037	0.047	0.043	0.056	0.053
0.039	0.037	0.037	0.039	0.040	0.037	0.044	0.044	0.048	0.039	0.038	0.034	0.012	0.034	0.052	0.049
0.041	0.039	0.039	0.041	0.041	0.039	0.046	0.046	0.049	0.040	0.040	0.036	0.014	0.036	0.054	0.052
0.173	0.178	0.178	0.175	0.175	0.177	0.179	0.179	0.170	0.178	0.177	0.176	0.177	0.176	0.175	0.185
0.176	0.179	0.181	0.177	0.178	0.179	0.179	0.179	0.171	0.181	0.178	0.178	0.181	0.178	0.178	0.184
0.174	0.177	0.177	0.174	0.173	0.177	0.178	0.178	0.172	0.177	0.176	0.175	0.177	0.177	0.175	0.181
0.188	0.186	0.186	0.185	0.185	0.185	0.191	0.191	0.182	0.186	0.185	0.183	0.185	0.185	0.182	0.182
0.207	0.203	0.203	0.206	0.206	0.204	0.202	0.202	0.200	0.205	0.203	0.206	0.206	0.206	0.200	0.202
0.193	0.192	0.192	0.196	0.196	0.194	0.196	0.196	0.196	0.195	0.193	0.192	0.196	0.196	0.189	0.196
0.182	0.183	0.184	0.182	0.181	0.181	0.183	0.183	0.181	0.182	0.181	0.184	0.185	0.184	0.186	0.184
0.046	0.044	0.044	0.047	0.047	0.047	0.051	0.051	0.051	0.049	0.047	0.041	0.040	0.045	0.046	0.046
0.032	0.030	0.030	0.034	0.034	0.034	0.034	0.034	0.041	0.036	0.035	0.027	0.036	0.033	0.037	0.039
0.031	0.031	0.031	0.032	0.033	0.033	0.034	0.034	0.042	0.035	0.034	0.027	0.032	0.032	0.033	0.036
0.179	0.181	0.182	0.181	0.179	0.180	0.185	0.185	0.178	0.181	0.181	0.181	0.177	0.180	0.180	0.178
0.024	0.022	0.022	0.025	0.026	0.020	0.027	0.027	0.035	0.022	0.022	0.018	0.030	0.013	0.048	0.044
0.023	0.020	0.020	0.026	0.026	0.021	0.028	0.028	0.036	0.022	0.022	0.018	0.032	0.014	0.049	0.048
0.296	0.293	0.294	0.296	0.295	0.294	0.295	0.295	0.295	0.296	0.294	0.292	0.294	0.293	0.295	0.295
0.174	0.175	0.176	0.169	0.168	0.172	0.174	0.174	0.170	0.172	0.172	0.175	0.167	0.172	0.165	0.171
0.182	0.184	0.185	0.181	0.182	0.182	0.185	0.185	0.179	0.183	0.184	0.185	0.182	0.184	0.188	0.186
0.177	0.178	0.179	0.179	0.179	0.175	0.177	0.177	0.170	0.175	0.176	0.179	0.179	0.181	0.181	0.181

87	88	89	90	91	92	93	94	95	96	97	98	99	100	101	102

0.044															
0.012	0.038														
0.034	0.046	0.028													
0.053	0.046	0.046	0.052												
0.053	0.039	0.048	0.054	0.058											
0.049	0.042	0.041	0.051	0.021	0.051										
0.052	0.044	0.044	0.053	0.023	0.053	0.004									
0.185	0.176	0.181	0.175	0.176	0.178	0.179	0.178								
0.184	0.179	0.184	0.179	0.179	0.181	0.183	0.182	0.022							
0.181	0.175	0.178	0.176	0.177	0.179	0.179	0.179	0.026	0.033						
0.182	0.190	0.182	0.180	0.185	0.190	0.187	0.186	0.157	0.155	0.158					
0.202	0.199	0.205	0.200	0.208	0.198	0.206	0.207	0.194	0.200	0.201	0.207				
0.196	0.196	0.194	0.192	0.197	0.191	0.196	0.198	0.188	0.192	0.186	0.203	0.122			
0.184	0.182	0.182	0.185	0.186	0.178	0.184	0.183	0.163	0.160	0.158	0.164	0.207	0.194		
0.046	0.044	0.039	0.045	0.050	0.052	0.049	0.051	0.172	0.174	0.171	0.179	0.193	0.191	0.178	
0.039	0.034	0.032	0.037	0.047	0.043	0.044	0.046	0.177	0.179	0.175	0.179	0.200	0.189	0.182	0.035
0.036	0.034	0.029	0.033	0.041	0.041	0.040	0.041	0.178	0.179	0.176	0.182	0.196	0.190	0.182	0.030
0.178	0.178	0.176	0.179	0.177	0.172	0.178	0.178	0.168	0.164	0.161	0.165	0.200	0.196	0.057	0.168
0.044	0.036	0.041	0.047	0.039	0.043	0.035	0.037	0.177	0.178	0.177	0.185	0.205	0.198	0.186	0.049
0.048	0.036	0.044	0.048	0.041	0.044	0.037	0.039	0.178	0.181	0.179	0.192	0.203	0.197	0.186	0.051
0.295	0.290	0.297	0.295	0.296	0.290	0.292	0.291	0.312	0.308	0.306	0.302	0.288	0.276	0.301	0.291
0.171	0.165	0.167	0.166	0.165	0.167	0.169	0.169	0.156	0.153	0.153	0.160	0.199	0.186	0.111	0.163
0.186	0.182	0.184	0.187	0.181	0.178	0.182	0.182	0.161	0.158	0.160	0.165	0.203	0.196	0.046	0.177
0.181	0.179	0.180	0.180	0.179	0.176	0.183	0.180	0.146	0.147	0.146	0.155	0.196	0.182	0.144	0.173

103 104 105 106 107 108 109 110 111

0.016							
0.174	0.172						
0.037	0.036	0.181					
0.039	0.037	0.184	0.010				
0.293	0.294	0.297	0.295	0.292			
0.164	0.165	0.102	0.174	0.175	0.292		
0.179	0.181	0.053	0.186	0.188	0.293	0.089	
0.177	0.179	0.149	0.182	0.181	0.291	0.137	0.137

Table. Estimates of Evolutionary Divergence between Sequences

The number of base differences per site from between sequences are shown. The analysis involved 111 nucleotide sequences. Codon positions included were 1st+2nd+3rd+Noncoding. All positions containing gaps and missing data were eliminated. There were a total of 1711 positions in the final dataset. Evolutionary analyses were conducted in MEGA7 [1].

1. Kumar S., Stecher G., and Tamura K. (2016). MEGA7: Molecular Evolutionary Genetics Analysis version 7.0 for bigger datasets.Molecular Biology and Evolution 33:1870-1874.

Disclaimer: Although utmost care has been taken to ensure the correctness of the caption, the caption text is provided "as is" without any warranty of any kind. Authors advise the user to carefully check the caption prior to its use for any purpose and report any errors or problems to the authors immediately (www.megasoftware.net). In no event shall the authors and their employers be liable for any damages, including but not limited to special, consequential, or other damages. Authors specifically disclaim all other warranties expressed or implied, including but not limited to the determination of suitability of this caption text for a specific purpose, use, or application.
