# Supplementary material for: Simultaneous Occurrence of Field Epidemics of Rabbit Hemorrhagic Disease (RHD) in Poland Due to the Co-Presence of Lagovirus europaeus GI.1 (RHDV)/GI.1a (RHDVa) and GI.2 (RHDV2) Genotypes
Source: Viruses. 2025 Sep 26;17(10):1305. doi: 10.3390/v17101305 (PMC12568209; doi:10.3390/v17101305)
Supplement: Supplementary file 1 [file viruses-17-01305-s001.zip › Tab S3 108 seqs NS genetic distance (1).pdf]

	108 sekw NSP	1	2	3	4	5	6
1	WAE_2022_(OR488784)_PL						
2	ZWO_2021_(OQ605827)_PL	0.095					
3	KOB_2020_(OQ605828)_PL	0.016	0.092				
4	LIB_2020_(OQ605829)_PL	0.154	0.153	0.150			
5	NRU_2020_(OQ605830)_PL	0.155	0.150	0.151	0.026		
6	PD_1989_(KP144789)_PL	0.136	0.136	0.133	0.117	0.117	
7	MAL_1994_(KU882093)_PL	0.139	0.139	0.136	0.120	0.119	0.011
8	BLA_1994_(KP144792)_PL	0.137	0.139	0.135	0.120	0.121	0.058
9	OPO_2004_(KU882094)_PL	0.139	0.140	0.136	0.122	0.122	0.060
10	GSK_1998_(KU882092)_PL	0.139	0.140	0.136	0.120	0.121	0.064
11	ZD0_2000_(KU882095)_PL	0.141	0.142	0.137	0.122	0.121	0.064
12	GRZ_2004_(KP144791)_PL	0.153	0.151	0.148	0.019	0.021	0.114
13	L14504_2004_(KY679902)_PL	0.153	0.151	0.149	0.017	0.020	0.114
14	W14705_2005_(KY319035)_PL	0.152	0.151	0.148	0.019	0.020	0.113
15	STR_2012_(KF677011)_PL	0.155	0.154	0.152	0.036	0.036	0.116
16	GLE_2013_(KY319032)_PL	0.157	0.153	0.152	0.009	0.025	0.117
17	SKO_2013_(KY319034)_PL	0.155	0.154	0.153	0.036	0.037	0.116
18	RED1_2013_(KY679903)_PL	0.151	0.150	0.148	0.045	0.047	0.115
19	STR2_2013_(KY679904)_PL	0.155	0.153	0.150	0.024	0.025	0.119
20	STR_2014_(KY679905)_PL	0.156	0.154	0.154	0.037	0.038	0.117
21	BIE_2015_(KY319031)_PL	0.156	0.154	0.153	0.036	0.036	0.117
22	F77-3_2015_(MN853658)_PL	0.153	0.152	0.151	0.046	0.045	0.116
23	BBI_2017_(MG602005)_PL	0.154	0.152	0.150	0.025	0.027	0.118
24	RED_2016_(MG602006)_PL	0.154	0.154	0.148	0.133	0.133	0.101
25	VMS_2017_(MG602007)_PL	0.154	0.153	0.148	0.134	0.133	0.098
26	PIN_2018_(MN853660)_PL	0.090	0.092	0.088	0.153	0.150	0.134
27	LIB_2018_(MN853659)_PL	0.012	0.092	0.006	0.149	0.150	0.133
28	WAK_2018_(MN853661)_PL	0.091	0.094	0.088	0.153	0.151	0.135
29	V-351_1987(U54983)_CZ	0.140	0.139	0.137	0.122	0.123	0.013
30	FRG_1989_(M67473)_DE	0.136	0.137	0.133	0.116	0.116	0.007
31	Jena_1993_(EF558576)_DE	0.138	0.142	0.134	0.117	0.117	0.061
32	Frankfurt5_1996_(EF558573)_DE	0.139	0.139	0.137	0.121	0.122	0.060
33	AST89_1989_(Z49271)_ES	0.138	0.137	0.133	0.117	0.117	0.057
34	RHDV-SD_1989_(Z29514)_FR	0.139	0.140	0.135	0.119	0.119	0.060
35	95-10_1995_(MT628287)_FR	0.141	0.139	0.138	0.120	0.121	0.062
36	00-21_2000_(MH190418)_FR	0.137	0.140	0.133	0.122	0.124	0.069
37	09-02_2009_(MT628289)_FR	0.136	0.139	0.134	0.117	0.118	0.060
38	09-03_2009_(MT628290)_FR	0.080	0.082	0.077	0.152	0.151	0.130
39	96VLT000113_1995_(MT819374)_S	0.140	0.142	0.138	0.123	0.122	0.066
40	12VLT000099_2012_(MT819377)_S	0.078	0.080	0.076	0.147	0.146	0.129
41	BS89_1989_(X87607)_IT	0.138	0.139	0.136	0.119	0.118	0.056
42	CB137_1995_(JX886002)_PT	0.141	0.141	0.136	0.126	0.127	0.070
43	CB156_1997_(JF438967)_PT	0.139	0.140	0.135	0.120	0.125	0.064
44	CB194_2006_(JX886001)_PT	0.146	0.146	0.142	0.132	0.134	0.086
45	Woodcroft_2005_(KT006741)_AUS	0.141	0.143	0.137	0.125	0.123	0.033
46	Triptis_1996_(EF558583)_DE	0.152	0.150	0.148	0.033	0.034	0.111
47	Erfurt_1996_(EF558581)_DE	0.152	0.150	0.148	0.020	0.020	0.114
48	Rossi_2002_(EF558584)_DE	0.152	0.149	0.148	0.039	0.041	0.114
49	P175_1999_(KY622129)_PT	0.151	0.151	0.149	0.044	0.046	0.113
50	RHDV-Hokkaido_2002_(AB300693)_JPN	0.154	0.153	0.151	0.049	0.051	0.119
51	WHNRH_2005_(DQ280493)_CN	0.153	0.150	0.149	0.024	0.023	0.115
52	RHDV_2014_(MK895974)_CN	0.152	0.152	0.149	0.047	0.045	0.115
53	WIN-AH-2011-OTH-026_(KY235676)_CAN	0.153	0.153	0.150	0.042	0.043	0.116
54	Iowa_2000_(AF258618)_USA	0.154	0.152	0.151	0.041	0.043	0.116
55	IN-05_2005_(EU003578)_USA	0.151	0.149	0.146	0.030	0.026	0.115

56	13-165_2013_(MN737112)_FR	0.151	0.147	0.145	0.132	0.131	0.096
57	16-35OOd_2016_(MN738377)_FR	0.020	0.088	0.015	0.147	0.147	0.132
58	NL-2016_(MN061492)_NL	0.090	0.092	0.087	0.153	0.150	0.135
59	N11_2011_(KM878681)_ES	0.085	0.035	0.084	0.149	0.147	0.134
60	Zar11-11_2011_(KP129398)_ES	0.083	0.035	0.082	0.147	0.146	0.134
61	CBAnd1_2012_(KP090976)_ES	0.229	0.231	0.229	0.232	0.226	0.230
62	Seg08-12_2012_(KP129396)_ES	0.086	0.038	0.084	0.148	0.146	0.133
63	Rij06-12_2012_(KP129395)_ES	0.085	0.038	0.083	0.148	0.146	0.133
64	16PLM1_2016_(MF407653)_ES	0.091	0.049	0.088	0.151	0.150	0.137
65	CBVal16_2012_(KM979445)_PT	0.086	0.039	0.084	0.148	0.146	0.134
66	Algarve1_2013_(KF442961)_PT	0.228	0.233	0.227	0.232	0.228	0.232
67	7-13_Barrancos_2013_(KF442963)_PT	0.229	0.234	0.228	0.232	0.227	0.232
68	10A-13_Barrancos_2013_(KF442964)_PT	0.229	0.234	0.228	0.231	0.227	0.232
69	CBAlgarve14-1_2014_(KM115714)_PT	0.153	0.149	0.147	0.130	0.132	0.098
70	CBAlgarve14-3_2014_(KM115715)_PT	0.153	0.149	0.146	0.131	0.132	0.098
71	CBEstremoz14-1_2014_(KM115681)_PT	0.228	0.233	0.227	0.232	0.228	0.232
72	CBMert_14-1_2014_(KM115712)_PT	0.153	0.149	0.146	0.131	0.133	0.099
73	CBMert14-2_2014_(KM115713)_PT	0.153	0.149	0.147	0.131	0.133	0.098
74	SOS089_2014_(MG763936)_PT	0.154	0.152	0.148	0.132	0.133	0.101
75	SOS158_2015_(MG763947)_PT	0.154	0.153	0.148	0.132	0.133	0.101
76	PSM2_2016_(MF407654)_PT	0.158	0.153	0.152	0.134	0.135	0.105
77	CBPico17-1_2017_(MF407651)_PT	0.157	0.155	0.151	0.136	0.138	0.107
78	CBPico17-2_2017_(MF407652)_PT	0.157	0.155	0.151	0.135	0.137	0.108
79	CBMad17-1_2017_(MF407655)_PT	0.230	0.232	0.229	0.229	0.225	0.230
80	CBMad17-2_2017_(MF407656)_PT	0.158	0.153	0.152	0.134	0.134	0.106
81	CBMad17-3_2017_(MF407657)_PT	0.157	0.153	0.151	0.134	0.135	0.105
82	RHDV/GER-NW/D51-1.L00911_2014_(LR899189)_DE	0.155	0.153	0.149	0.132	0.133	0.100
83	EI327.L03607/2016_(LR899157)_DE	0.090	0.090	0.086	0.153	0.150	0.134
84	BLMT-1_2015_(KT280060)_AUS	0.156	0.152	0.149	0.133	0.135	0.100
85	AZ1_2020_(MT506237)_USA	0.035	0.088	0.032	0.148	0.147	0.131
86	NY1_2020_(MT506236)_USA	0.037	0.091	0.033	0.149	0.149	0.133
87	NY2_2020_(MT506235)_USA	0.038	0.091	0.034	0.148	0.148	0.133
88	WIN-AH-2016-OTH-0018_(KY235675)_CAN	0.086	0.048	0.085	0.146	0.146	0.133
89	WIN-AH-2019-OTH-0032_(MT900574)_CAN	0.039	0.090	0.035	0.150	0.152	0.133
90	Senasica20_2020_(OM973948)_MX	0.034	0.088	0.031	0.148	0.148	0.132
91	RHDV2-S25_2019_(MW789242)_GH	0.093	0.094	0.088	0.154	0.152	0.134
92	Touza_1_2019_(MZ913394)_TN	0.093	0.041	0.089	0.151	0.149	0.137
93	Ibaraki-1_2019_(LC749423)_JPN	0.089	0.091	0.087	0.151	0.149	0.133
94	Chiba-1_2020_(LC749425)_JPN	0.090	0.091	0.088	0.152	0.149	0.132
95	06-11_2006_(MN737115)_FR	0.068	0.074	0.065	0.144	0.143	0.126
96	08-133_2008_(MN746289)_FR	0.073	0.077	0.069	0.142	0.143	0.128
97	09-48_2009_(MN737116)_FR	0.071	0.078	0.070	0.144	0.143	0.130
98	MRCV_2001_(GQ166866)_USA	0.220	0.219	0.216	0.221	0.218	0.217
99	RCV-A1_MIC-07_2007_(EU871528)_AUS	0.220	0.223	0.219	0.224	0.221	0.223
100	Australia-1_RCV_2007_(KX357690)_AUS	0.230	0.228	0.227	0.232	0.227	0.223
101	AUS/NSW/ANN-1/2014/04_(KY628306)_AUS	0.221	0.222	0.219	0.222	0.223	0.222
102	Otago/NZ-37/2018_(OM372665)_NZ	0.091	0.093	0.088	0.148	0.146	0.133
103	10-28_2010_(MN737113)_FR	0.078	0.085	0.074	0.146	0.145	0.129
104	10-32_2010_(MN737114)_FR	0.085	0.088	0.081	0.148	0.147	0.132
105	K5_08Q712_2008_(MF598301)_AUS	0.155	0.154	0.152	0.057	0.058	0.124
106	ACT/AIN-5_2017_(MW460019)_AUS	0.223	0.223	0.221	0.221	0.222	0.222
107	NSW/CAR-3/2016_(MF598302)_AUS	0.222	0.222	0.220	0.221	0.222	0.220
108	EBHSV-GD_(Z69620)_FR	0.281	0.279	0.281	0.289	0.289	0.281

7	8	9	10	11	12	13	14	15	16	17	18	19	20	21	22
0.062															
0.064	0.007														
0.069	0.033	0.036													
0.069	0.033	0.037	0.022												
0.116	0.117	0.119	0.119	0.117											
0.116	0.116	0.118	0.118	0.118	0.009										
0.116	0.116	0.118	0.118	0.116	0.003	0.008									
0.118	0.118	0.121	0.120	0.120	0.031	0.029	0.031								
0.119	0.120	0.121	0.120	0.122	0.017	0.015	0.017	0.033							
0.119	0.120	0.122	0.121	0.121	0.032	0.029	0.032	0.006	0.034						
0.117	0.118	0.119	0.120	0.117	0.042	0.041	0.042	0.045	0.045	0.046					
0.120	0.120	0.122	0.122	0.122	0.018	0.017	0.017	0.034	0.020	0.035	0.045				
0.118	0.120	0.122	0.120	0.121	0.032	0.031	0.033	0.006	0.035	0.004	0.047	0.036			
0.118	0.120	0.122	0.121	0.122	0.031	0.029	0.031	0.005	0.033	0.003	0.045	0.034	0.002		
0.118	0.120	0.121	0.121	0.120	0.041	0.039	0.040	0.040	0.044	0.040	0.054	0.044	0.040	0.039	
0.121	0.120	0.122	0.122	0.122	0.015	0.015	0.014	0.038	0.023	0.038	0.046	0.024	0.039	0.038	0.048
0.103	0.091	0.093	0.098	0.098	0.129	0.129	0.129	0.133	0.132	0.134	0.130	0.134	0.134	0.134	0.139
0.101	0.092	0.094	0.096	0.096	0.129	0.130	0.129	0.134	0.132	0.135	0.130	0.134	0.135	0.135	0.139
0.137	0.138	0.140	0.141	0.141	0.151	0.151	0.151	0.155	0.153	0.154	0.150	0.153	0.155	0.154	0.150
0.135	0.134	0.135	0.136	0.137	0.148	0.148	0.148	0.150	0.152	0.151	0.147	0.150	0.152	0.151	0.148
0.138	0.138	0.139	0.139	0.140	0.153	0.152	0.151	0.155	0.154	0.154	0.150	0.153	0.155	0.155	0.151
0.017	0.063	0.065	0.068	0.069	0.120	0.119	0.119	0.121	0.122	0.121	0.120	0.124	0.121	0.121	0.121
0.011	0.059	0.061	0.064	0.065	0.113	0.113	0.112	0.115	0.116	0.115	0.115	0.118	0.115	0.115	0.115
0.065	0.028	0.031	0.018	0.015	0.114	0.113	0.113	0.116	0.117	0.117	0.115	0.117	0.117	0.117	0.117
0.063	0.006	0.011	0.037	0.037	0.118	0.117	0.117	0.120	0.121	0.121	0.119	0.121	0.121	0.121	0.121
0.060	0.044	0.047	0.050	0.050	0.112	0.112	0.111	0.114	0.117	0.115	0.114	0.116	0.115	0.114	0.118
0.063	0.048	0.051	0.052	0.052	0.114	0.113	0.113	0.114	0.119	0.115	0.115	0.119	0.115	0.115	0.120
0.066	0.022	0.026	0.037	0.038	0.117	0.117	0.116	0.120	0.120	0.120	0.120	0.121	0.120	0.121	0.119
0.072	0.041	0.044	0.029	0.032	0.119	0.119	0.118	0.122	0.122	0.122	0.119	0.124	0.122	0.123	0.125
0.064	0.025	0.028	0.032	0.034	0.113	0.113	0.112	0.116	0.117	0.117	0.115	0.118	0.118	0.118	0.118
0.132	0.135	0.136	0.139	0.138	0.150	0.149	0.149	0.152	0.152	0.153	0.152	0.149	0.153	0.153	0.153
0.071	0.036	0.040	0.026	0.017	0.120	0.120	0.119	0.121	0.123	0.122	0.119	0.124	0.122	0.122	0.123
0.133	0.133	0.133	0.134	0.135	0.145	0.145	0.145	0.148	0.147	0.149	0.146	0.147	0.149	0.148	0.149
0.059	0.018	0.021	0.029	0.030	0.115	0.114	0.114	0.116	0.118	0.117	0.116	0.119	0.117	0.117	0.116
0.071	0.056	0.058	0.064	0.063	0.123	0.121	0.122	0.124	0.125	0.124	0.122	0.127	0.125	0.125	0.126
0.067	0.054	0.056	0.061	0.061	0.118	0.118	0.117	0.121	0.121	0.121	0.120	0.123	0.122	0.121	0.124
0.089	0.072	0.075	0.081	0.082	0.128	0.128	0.127	0.131	0.130	0.132	0.128	0.133	0.132	0.131	0.133
0.037	0.072	0.074	0.078	0.078	0.123	0.122	0.122	0.123	0.124	0.124	0.122	0.126	0.124	0.124	0.124
0.112	0.112	0.113	0.116	0.113	0.029	0.028	0.029	0.033	0.032	0.034	0.021	0.032	0.035	0.033	0.042
0.115	0.115	0.117	0.117	0.117	0.015	0.014	0.015	0.025	0.018	0.026	0.038	0.020	0.027	0.025	0.036
0.114	0.115	0.116	0.119	0.116	0.036	0.035	0.036	0.040	0.038	0.041	0.029	0.038	0.042	0.040	0.048
0.115	0.116	0.117	0.119	0.116	0.041	0.039	0.040	0.045	0.044	0.044	0.025	0.044	0.046	0.044	0.051
0.120	0.119	0.120	0.122	0.119	0.046	0.045	0.045	0.050	0.049	0.050	0.032	0.049	0.051	0.050	0.056
0.117	0.119	0.120	0.120	0.120	0.019	0.018	0.019	0.035	0.023	0.035	0.045	0.024	0.036	0.034	0.044
0.116	0.119	0.121	0.120	0.119	0.041	0.039	0.041	0.040	0.045	0.039	0.054	0.044	0.040	0.040	0.019
0.118	0.121	0.123	0.122	0.122	0.038	0.036	0.037	0.035	0.040	0.035	0.050	0.042	0.036	0.035	0.043
0.117	0.117	0.119	0.119	0.117	0.038	0.037	0.038	0.041	0.041	0.042	0.024	0.040	0.043	0.041	0.050
0.117	0.117	0.118	0.119	0.117	0.025	0.024	0.025	0.037	0.029	0.038	0.046	0.030	0.039	0.037	0.046

0.098	0.086	0.088	0.092	0.091	0.129	0.128	0.128	0.131	0.131	0.132	0.129	0.132	0.132	0.132	0.135
0.134	0.132	0.134	0.134	0.136	0.146	0.146	0.146	0.148	0.148	0.148	0.144	0.147	0.149	0.148	0.148
0.137	0.139	0.141	0.141	0.141	0.152	0.151	0.151	0.154	0.153	0.153	0.151	0.152	0.154	0.154	0.149
0.137	0.139	0.140	0.138	0.141	0.148	0.148	0.148	0.151	0.149	0.151	0.150	0.150	0.151	0.150	0.151
0.137	0.140	0.141	0.139	0.141	0.148	0.147	0.147	0.150	0.148	0.150	0.150	0.149	0.150	0.150	0.150
0.231	0.227	0.228	0.229	0.227	0.228	0.228	0.228	0.228	0.232	0.229	0.226	0.231	0.229	0.228	0.231
0.136	0.139	0.140	0.138	0.141	0.148	0.147	0.148	0.150	0.148	0.150	0.150	0.149	0.150	0.150	0.150
0.136	0.139	0.140	0.138	0.140	0.148	0.147	0.148	0.150	0.148	0.150	0.149	0.150	0.150	0.149	0.150
0.141	0.142	0.143	0.141	0.143	0.152	0.151	0.151	0.153	0.152	0.154	0.151	0.152	0.153	0.154	0.153
0.137	0.141	0.142	0.140	0.142	0.148	0.147	0.148	0.151	0.148	0.151	0.150	0.149	0.151	0.151	0.150
0.233	0.230	0.229	0.228	0.229	0.230	0.229	0.229	0.229	0.232	0.228	0.229	0.231	0.228	0.228	0.230
0.233	0.230	0.229	0.228	0.229	0.230	0.228	0.229	0.228	0.231	0.228	0.228	0.231	0.227	0.227	0.229
0.232	0.229	0.228	0.227	0.228	0.229	0.227	0.228	0.228	0.231	0.227	0.227	0.230	0.226	0.226	0.229
0.100	0.090	0.092	0.095	0.094	0.126	0.127	0.127	0.131	0.130	0.132	0.129	0.132	0.133	0.132	0.136
0.100	0.090	0.092	0.095	0.095	0.127	0.128	0.127	0.132	0.130	0.132	0.129	0.132	0.133	0.133	0.136
0.232	0.230	0.229	0.228	0.229	0.231	0.229	0.230	0.229	0.232	0.229	0.229	0.231	0.229	0.228	0.230
0.101	0.090	0.092	0.095	0.095	0.128	0.128	0.128	0.132	0.131	0.133	0.130	0.133	0.134	0.133	0.137
0.100	0.090	0.092	0.095	0.095	0.128	0.128	0.128	0.131	0.131	0.132	0.129	0.132	0.133	0.132	0.136
0.102	0.092	0.093	0.098	0.097	0.128	0.129	0.128	0.133	0.131	0.134	0.131	0.133	0.135	0.134	0.138
0.103	0.092	0.094	0.098	0.097	0.128	0.129	0.128	0.133	0.132	0.134	0.131	0.133	0.135	0.134	0.137
0.106	0.095	0.097	0.101	0.100	0.130	0.130	0.130	0.134	0.133	0.135	0.133	0.134	0.136	0.135	0.139
0.109	0.097	0.098	0.101	0.101	0.132	0.133	0.132	0.139	0.136	0.139	0.134	0.137	0.139	0.138	0.140
0.109	0.097	0.098	0.101	0.102	0.132	0.132	0.132	0.138	0.136	0.138	0.134	0.136	0.138	0.138	0.139
0.232	0.229	0.228	0.227	0.228	0.229	0.226	0.228	0.228	0.229	0.227	0.228	0.229	0.226	0.226	0.229
0.107	0.095	0.098	0.102	0.101	0.129	0.130	0.129	0.134	0.133	0.135	0.133	0.134	0.136	0.135	0.139
0.106	0.095	0.097	0.101	0.100	0.130	0.130	0.129	0.134	0.133	0.135	0.133	0.134	0.136	0.135	0.139
0.102	0.092	0.094	0.098	0.097	0.128	0.129	0.129	0.133	0.131	0.134	0.131	0.134	0.135	0.134	0.138
0.137	0.139	0.140	0.140	0.140	0.152	0.151	0.151	0.155	0.153	0.154	0.150	0.153	0.155	0.154	0.150
0.102	0.090	0.092	0.097	0.097	0.130	0.130	0.129	0.134	0.133	0.136	0.131	0.134	0.136	0.135	0.137
0.134	0.130	0.132	0.134	0.135	0.146	0.146	0.146	0.148	0.148	0.149	0.146	0.147	0.149	0.149	0.148
0.136	0.134	0.136	0.137	0.138	0.148	0.148	0.147	0.149	0.150	0.149	0.146	0.149	0.150	0.149	0.148
0.136	0.134	0.136	0.136	0.137	0.147	0.147	0.146	0.148	0.149	0.149	0.146	0.148	0.149	0.149	0.147
0.136	0.137	0.138	0.137	0.139	0.147	0.146	0.146	0.149	0.147	0.149	0.150	0.148	0.149	0.149	0.148
0.136	0.135	0.137	0.137	0.139	0.150	0.150	0.149	0.152	0.151	0.153	0.149	0.151	0.153	0.153	0.150
0.135	0.132	0.134	0.136	0.137	0.147	0.147	0.147	0.148	0.149	0.149	0.146	0.148	0.150	0.149	0.149
0.137	0.140	0.141	0.141	0.142	0.153	0.152	0.152	0.156	0.154	0.156	0.153	0.155	0.157	0.156	0.151
0.141	0.140	0.141	0.142	0.145	0.150	0.148	0.148	0.151	0.150	0.152	0.151	0.151	0.152	0.152	0.153
0.135	0.137	0.138	0.138	0.139	0.151	0.151	0.150	0.154	0.152	0.153	0.149	0.152	0.154	0.154	0.149
0.134	0.136	0.137	0.138	0.139	0.152	0.150	0.151	0.154	0.152	0.153	0.150	0.152	0.154	0.154	0.149
0.130	0.130	0.130	0.132	0.134	0.143	0.142	0.142	0.146	0.144	0.146	0.143	0.145	0.146	0.146	0.146
0.132	0.132	0.133	0.132	0.135	0.142	0.142	0.141	0.146	0.143	0.147	0.143	0.144	0.147	0.147	0.145
0.134	0.133	0.134	0.134	0.136	0.142	0.142	0.142	0.146	0.144	0.147	0.143	0.144	0.147	0.147	0.146
0.217	0.216	0.217	0.220	0.221	0.219	0.217	0.217	0.219	0.221	0.220	0.219	0.219	0.221	0.219	0.220
0.223	0.218	0.220	0.218	0.219	0.221	0.220	0.220	0.222	0.222	0.222	0.218	0.221	0.222	0.222	0.225
0.224	0.222	0.223	0.221	0.224	0.229	0.228	0.228	0.231	0.232	0.232	0.229	0.230	0.232	0.232	0.231
0.222	0.220	0.222	0.226	0.224	0.224	0.223	0.223	0.224	0.223	0.225	0.226	0.223	0.225	0.225	0.223
0.136	0.135	0.137	0.138	0.138	0.148	0.148	0.147	0.152	0.149	0.152	0.148	0.150	0.152	0.152	0.151
0.133	0.132	0.133	0.134	0.134	0.143	0.142	0.143	0.147	0.147	0.148	0.144	0.146	0.148	0.148	0.150
0.134	0.135	0.136	0.136	0.136	0.147	0.145	0.146	0.149	0.148	0.149	0.147	0.147	0.149	0.149	0.148
0.125	0.123	0.122	0.126	0.123	0.053	0.053	0.053	0.059	0.055	0.059	0.043	0.057	0.060	0.059	0.067
0.222	0.221	0.223	0.226	0.224	0.222	0.221	0.221	0.222	0.222	0.223	0.225	0.222	0.222	0.223	0.222
0.222	0.220	0.223	0.224	0.222	0.223	0.222	0.221	0.223	0.222	0.224	0.226	0.223	0.224	0.224	0.224
0.283	0.279	0.279	0.283	0.283	0.288	0.287	0.288	0.292	0.287	0.292	0.288	0.289	0.292	0.292	0.287

23	24	25	26	27	28	29	30	31	32	33	34	35	36	37	38
0.133															
0.133	0.026														
0.152	0.150	0.153													
0.150	0.149	0.150	0.087												
0.153	0.150	0.152	0.018	0.087											
0.124	0.105	0.103	0.139	0.136	0.140										
0.118	0.100	0.098	0.134	0.132	0.135	0.011									
0.118	0.096	0.093	0.140	0.134	0.139	0.065	0.061								
0.122	0.093	0.095	0.139	0.136	0.139	0.065	0.061	0.031							
0.116	0.075	0.074	0.138	0.134	0.137	0.061	0.057	0.045	0.047						
0.117	0.078	0.077	0.141	0.135	0.140	0.064	0.060	0.049	0.051	0.011					
0.121	0.094	0.094	0.139	0.137	0.137	0.067	0.063	0.032	0.025	0.047	0.051				
0.123	0.097	0.095	0.140	0.133	0.139	0.073	0.069	0.028	0.045	0.054	0.057	0.047			
0.118	0.093	0.092	0.137	0.133	0.136	0.064	0.060	0.029	0.029	0.042	0.044	0.031	0.038		
0.151	0.149	0.149	0.083	0.076	0.085	0.133	0.130	0.137	0.138	0.135	0.136	0.139	0.135	0.133	
0.124	0.098	0.096	0.139	0.137	0.139	0.071	0.067	0.019	0.040	0.051	0.054	0.041	0.034	0.036	0.137
0.147	0.148	0.149	0.085	0.074	0.085	0.134	0.130	0.133	0.135	0.132	0.132	0.135	0.132	0.131	0.047
0.119	0.093	0.092	0.138	0.135	0.136	0.060	0.056	0.024	0.022	0.040	0.044	0.024	0.039	0.021	0.135
0.125	0.082	0.079	0.139	0.137	0.139	0.074	0.070	0.059	0.058	0.027	0.032	0.061	0.068	0.058	0.135
0.122	0.080	0.077	0.139	0.136	0.140	0.069	0.065	0.058	0.056	0.021	0.027	0.057	0.065	0.052	0.134
0.130	0.058	0.056	0.151	0.144	0.148	0.089	0.086	0.078	0.075	0.053	0.056	0.078	0.084	0.073	0.144
0.127	0.109	0.108	0.139	0.138	0.140	0.034	0.033	0.075	0.074	0.072	0.075	0.077	0.082	0.077	0.133
0.034	0.126	0.127	0.149	0.147	0.149	0.116	0.110	0.110	0.113	0.110	0.111	0.113	0.115	0.109	0.150
0.022	0.129	0.130	0.150	0.147	0.150	0.118	0.112	0.112	0.116	0.111	0.112	0.115	0.118	0.112	0.149
0.041	0.129	0.129	0.152	0.147	0.152	0.118	0.113	0.113	0.116	0.114	0.115	0.116	0.117	0.112	0.151
0.045	0.131	0.131	0.154	0.146	0.152	0.117	0.111	0.114	0.117	0.115	0.114	0.116	0.119	0.112	0.152
0.050	0.131	0.133	0.155	0.149	0.154	0.123	0.118	0.116	0.120	0.117	0.117	0.119	0.121	0.115	0.156
0.026	0.131	0.131	0.148	0.148	0.149	0.120	0.114	0.116	0.120	0.115	0.117	0.119	0.123	0.117	0.149
0.048	0.137	0.136	0.150	0.146	0.150	0.119	0.114	0.116	0.120	0.117	0.119	0.119	0.123	0.118	0.150
0.044	0.136	0.135	0.151	0.148	0.151	0.122	0.115	0.118	0.121	0.116	0.117	0.119	0.123	0.117	0.149
0.043	0.134	0.134	0.152	0.150	0.151	0.120	0.115	0.114	0.118	0.117	0.118	0.120	0.120	0.114	0.153
0.032	0.132	0.132	0.151	0.146	0.150	0.120	0.114	0.115	0.118	0.114	0.116	0.117	0.119	0.115	0.149

0.132	0.037	0.037	0.151	0.146	0.152	0.100	0.095	0.088	0.089	0.069	0.070	0.091	0.093	0.087	0.147
0.148	0.148	0.148	0.085	0.013	0.085	0.136	0.131	0.134	0.134	0.133	0.134	0.136	0.132	0.131	0.073
0.153	0.150	0.152	0.014	0.087	0.015	0.140	0.135	0.140	0.141	0.137	0.140	0.138	0.139	0.136	0.084
0.148	0.149	0.148	0.083	0.082	0.085	0.139	0.135	0.140	0.139	0.136	0.139	0.140	0.139	0.138	0.075
0.147	0.149	0.148	0.083	0.080	0.085	0.138	0.134	0.141	0.140	0.137	0.139	0.140	0.138	0.138	0.073
0.228	0.232	0.230	0.229	0.227	0.231	0.234	0.232	0.229	0.228	0.226	0.228	0.229	0.228	0.229	0.231
0.146	0.149	0.149	0.084	0.082	0.086	0.137	0.133	0.140	0.139	0.135	0.138	0.139	0.139	0.137	0.073
0.147	0.147	0.147	0.083	0.082	0.085	0.137	0.134	0.139	0.139	0.135	0.137	0.139	0.138	0.137	0.073
0.151	0.152	0.151	0.087	0.087	0.089	0.141	0.138	0.143	0.142	0.138	0.140	0.141	0.140	0.139	0.080
0.147	0.148	0.148	0.084	0.083	0.086	0.139	0.135	0.141	0.141	0.137	0.140	0.142	0.140	0.139	0.074
0.228	0.236	0.238	0.232	0.227	0.232	0.235	0.232	0.230	0.230	0.232	0.233	0.228	0.230	0.229	0.229
0.228	0.235	0.237	0.233	0.227	0.233	0.235	0.232	0.230	0.230	0.232	0.233	0.229	0.230	0.229	0.229
0.227	0.234	0.236	0.233	0.227	0.233	0.234	0.230	0.230	0.229	0.232	0.232	0.228	0.230	0.228	0.229
0.131	0.017	0.017	0.152	0.148	0.151	0.103	0.098	0.092	0.092	0.070	0.073	0.092	0.095	0.090	0.146
0.131	0.018	0.017	0.152	0.148	0.151	0.103	0.098	0.092	0.093	0.070	0.073	0.092	0.095	0.090	0.146
0.229	0.235	0.236	0.230	0.226	0.230	0.235	0.231	0.230	0.230	0.232	0.233	0.229	0.230	0.230	0.228
0.132	0.018	0.017	0.152	0.148	0.151	0.103	0.099	0.093	0.093	0.071	0.074	0.093	0.095	0.090	0.146
0.132	0.018	0.017	0.152	0.148	0.152	0.103	0.098	0.092	0.093	0.071	0.074	0.092	0.095	0.090	0.147
0.132	0.023	0.024	0.152	0.149	0.151	0.105	0.101	0.094	0.094	0.073	0.076	0.095	0.097	0.092	0.149
0.132	0.023	0.023	0.153	0.150	0.152	0.105	0.101	0.095	0.094	0.072	0.076	0.095	0.098	0.092	0.150
0.133	0.030	0.029	0.156	0.154	0.155	0.109	0.105	0.099	0.097	0.077	0.080	0.097	0.100	0.096	0.151
0.136	0.032	0.032	0.155	0.152	0.154	0.110	0.107	0.099	0.099	0.077	0.082	0.099	0.100	0.096	0.151
0.135	0.032	0.032	0.155	0.152	0.154	0.110	0.107	0.099	0.100	0.078	0.082	0.099	0.100	0.096	0.152
0.228	0.235	0.235	0.234	0.228	0.233	0.233	0.229	0.230	0.229	0.232	0.233	0.227	0.229	0.229	0.230
0.133	0.030	0.029	0.156	0.154	0.155	0.110	0.105	0.099	0.098	0.078	0.081	0.098	0.100	0.096	0.152
0.133	0.030	0.029	0.156	0.153	0.154	0.109	0.105	0.099	0.097	0.078	0.081	0.097	0.099	0.096	0.151
0.133	0.021	0.021	0.155	0.151	0.155	0.105	0.100	0.095	0.094	0.074	0.077	0.095	0.098	0.093	0.150
0.153	0.149	0.152	0.013	0.086	0.014	0.139	0.134	0.140	0.140	0.137	0.140	0.138	0.138	0.136	0.083
0.134	0.023	0.023	0.154	0.150	0.153	0.104	0.100	0.094	0.092	0.072	0.075	0.094	0.097	0.091	0.149
0.147	0.148	0.147	0.084	0.029	0.085	0.134	0.132	0.133	0.132	0.133	0.134	0.134	0.132	0.129	0.075
0.150	0.149	0.150	0.084	0.031	0.085	0.137	0.133	0.136	0.136	0.134	0.137	0.138	0.133	0.132	0.075
0.149	0.148	0.149	0.085	0.032	0.086	0.136	0.133	0.135	0.136	0.134	0.136	0.137	0.133	0.131	0.076
0.148	0.147	0.145	0.089	0.083	0.090	0.138	0.134	0.138	0.138	0.135	0.137	0.138	0.137	0.135	0.077
0.152	0.149	0.149	0.085	0.032	0.085	0.137	0.133	0.136	0.137	0.135	0.137	0.138	0.134	0.133	0.076
0.149	0.150	0.149	0.084	0.028	0.084	0.136	0.132	0.134	0.134	0.134	0.136	0.135	0.133	0.130	0.075
0.155	0.151	0.154	0.017	0.089	0.018	0.139	0.134	0.141	0.141	0.137	0.140	0.139	0.140	0.137	0.084
0.150	0.152	0.152	0.092	0.090	0.092	0.142	0.138	0.143	0.140	0.138	0.140	0.143	0.140	0.139	0.084
0.153	0.149	0.151	0.015	0.087	0.018	0.137	0.132	0.139	0.138	0.137	0.140	0.135	0.137	0.134	0.083
0.152	0.148	0.150	0.018	0.088	0.021	0.136	0.131	0.139	0.138	0.136	0.139	0.135	0.137	0.134	0.085
0.144	0.148	0.147	0.057	0.064	0.062	0.131	0.127	0.131	0.131	0.131	0.132	0.130	0.129	0.128	0.057
0.143	0.148	0.148	0.064	0.068	0.066	0.131	0.127	0.132	0.134	0.132	0.133	0.133	0.129	0.130	0.062
0.145	0.152	0.151	0.064	0.069	0.067	0.134	0.130	0.133	0.134	0.132	0.135	0.132	0.131	0.131	0.064
0.219	0.221	0.225	0.216	0.217	0.216	0.221	0.217	0.219	0.217	0.218	0.220	0.216	0.220	0.216	0.218
0.221	0.223	0.223	0.222	0.219	0.219	0.225	0.222	0.219	0.220	0.218	0.220	0.217	0.217	0.221	0.217
0.228	0.229	0.225	0.233	0.229	0.232	0.224	0.222	0.223	0.222	0.225	0.227	0.222	0.225	0.223	0.230
0.223	0.226	0.227	0.223	0.219	0.223	0.224	0.221	0.224	0.222	0.221	0.223	0.223	0.223	0.222	0.224
0.149	0.153	0.152	0.034	0.087	0.034	0.138	0.133	0.136	0.137	0.136	0.138	0.134	0.137	0.133	0.083
0.145	0.145	0.146	0.078	0.073	0.081	0.133	0.130	0.133	0.134	0.131	0.132	0.133	0.131	0.131	0.067
0.148	0.148	0.146	0.086	0.079	0.087	0.136	0.132	0.134	0.137	0.135	0.137	0.136	0.135	0.134	0.068
0.057	0.134	0.137	0.156	0.152	0.154	0.128	0.122	0.121	0.124	0.122	0.123	0.125	0.127	0.119	0.156
0.221	0.226	0.228	0.222	0.222	0.222	0.224	0.221	0.224	0.222	0.221	0.222	0.224	0.223	0.222	0.225
0.222	0.225	0.226	0.223	0.221	0.223	0.223	0.220	0.223	0.222	0.221	0.222	0.222	0.222	0.222	0.225
0.288	0.286	0.283	0.278	0.281	0.277	0.285	0.282	0.282	0.282	0.282	0.281	0.281	0.282	0.281	0.280

39	40	41	42	43	44	45	46	47	48	49	50	51	52	53	54
0.135															
0.033	0.133														
0.062	0.133	0.056													
0.063	0.132	0.052	0.028												
0.083	0.143	0.074	0.058	0.056											
0.079	0.133	0.071	0.082	0.080	0.097										
0.116	0.145	0.110	0.118	0.116	0.124	0.118									
0.118	0.145	0.112	0.121	0.118	0.127	0.122	0.024								
0.118	0.147	0.113	0.123	0.121	0.128	0.121	0.013	0.031							
0.118	0.147	0.114	0.123	0.120	0.128	0.120	0.019	0.037	0.027						
0.122	0.151	0.116	0.124	0.122	0.131	0.124	0.025	0.041	0.033	0.021					
0.123	0.146	0.117	0.125	0.120	0.129	0.124	0.032	0.020	0.040	0.044	0.049				
0.122	0.148	0.116	0.126	0.124	0.132	0.123	0.042	0.037	0.048	0.051	0.058	0.044			
0.123	0.145	0.118	0.124	0.122	0.132	0.126	0.039	0.032	0.045	0.047	0.053	0.042	0.043		
0.119	0.150	0.115	0.125	0.123	0.132	0.122	0.018	0.033	0.025	0.020	0.025	0.042	0.051	0.048	
0.120	0.145	0.115	0.123	0.121	0.129	0.123	0.035	0.023	0.042	0.045	0.051	0.029	0.046	0.043	0.043

0.092	0.143	0.088	0.073	0.073	0.051	0.103	0.124	0.128	0.128	0.129	0.131	0.131	0.132	0.133	0.133
0.136	0.072	0.133	0.136	0.135	0.144	0.137	0.144	0.144	0.144	0.145	0.147	0.146	0.147	0.147	0.147
0.140	0.086	0.137	0.139	0.139	0.150	0.139	0.149	0.149	0.152	0.153	0.154	0.148	0.150	0.150	0.152
0.141	0.073	0.139	0.137	0.138	0.146	0.140	0.147	0.147	0.148	0.149	0.152	0.148	0.150	0.150	0.150
0.141	0.071	0.139	0.138	0.139	0.146	0.140	0.147	0.146	0.147	0.149	0.151	0.147	0.149	0.149	0.149
0.225	0.229	0.227	0.226	0.228	0.230	0.231	0.229	0.229	0.230	0.228	0.229	0.230	0.229	0.230	0.225
0.140	0.072	0.138	0.136	0.137	0.146	0.139	0.148	0.146	0.149	0.149	0.151	0.147	0.150	0.151	0.149
0.139	0.072	0.138	0.136	0.137	0.146	0.139	0.147	0.146	0.148	0.149	0.151	0.148	0.150	0.149	0.149
0.141	0.079	0.141	0.140	0.140	0.150	0.143	0.151	0.150	0.152	0.152	0.153	0.152	0.153	0.152	0.151
0.141	0.074	0.141	0.138	0.139	0.146	0.140	0.147	0.147	0.148	0.149	0.151	0.147	0.151	0.150	0.150
0.227	0.225	0.228	0.233	0.232	0.239	0.234	0.229	0.229	0.231	0.226	0.228	0.230	0.228	0.230	0.227
0.226	0.226	0.228	0.232	0.231	0.238	0.234	0.229	0.228	0.231	0.226	0.227	0.230	0.228	0.230	0.227
0.226	0.225	0.227	0.232	0.230	0.238	0.234	0.228	0.228	0.230	0.225	0.227	0.229	0.227	0.229	0.226
0.095	0.144	0.091	0.076	0.074	0.051	0.108	0.124	0.128	0.127	0.130	0.131	0.129	0.134	0.134	0.132
0.095	0.144	0.092	0.077	0.074	0.051	0.107	0.125	0.128	0.128	0.130	0.131	0.129	0.134	0.134	0.133
0.227	0.225	0.228	0.232	0.231	0.238	0.234	0.229	0.229	0.231	0.227	0.227	0.231	0.229	0.231	0.227
0.096	0.144	0.092	0.077	0.074	0.052	0.108	0.125	0.129	0.128	0.131	0.132	0.130	0.134	0.135	0.133
0.095	0.145	0.092	0.076	0.074	0.052	0.107	0.125	0.129	0.128	0.130	0.132	0.130	0.134	0.134	0.132
0.097	0.146	0.094	0.079	0.077	0.055	0.110	0.127	0.129	0.129	0.132	0.134	0.131	0.136	0.135	0.134
0.097	0.148	0.094	0.079	0.077	0.055	0.110	0.127	0.129	0.129	0.132	0.134	0.131	0.136	0.135	0.135
0.102	0.150	0.097	0.083	0.082	0.059	0.113	0.128	0.131	0.130	0.132	0.134	0.132	0.138	0.136	0.135
0.102	0.150	0.098	0.085	0.082	0.062	0.114	0.131	0.133	0.134	0.135	0.137	0.135	0.138	0.140	0.137
0.103	0.150	0.098	0.086	0.082	0.063	0.115	0.130	0.133	0.134	0.135	0.136	0.135	0.138	0.139	0.136
0.227	0.227	0.228	0.232	0.231	0.237	0.233	0.229	0.228	0.231	0.226	0.226	0.228	0.227	0.227	0.227
0.102	0.151	0.097	0.084	0.082	0.059	0.113	0.127	0.130	0.130	0.132	0.134	0.132	0.138	0.136	0.135
0.102	0.150	0.097	0.084	0.082	0.059	0.113	0.128	0.131	0.130	0.132	0.134	0.132	0.138	0.136	0.135
0.098	0.149	0.094	0.079	0.077	0.055	0.109	0.126	0.129	0.129	0.132	0.134	0.131	0.136	0.134	0.134
0.140	0.084	0.137	0.138	0.139	0.150	0.139	0.149	0.149	0.152	0.154	0.155	0.148	0.150	0.150	0.152
0.098	0.148	0.092	0.079	0.077	0.055	0.109	0.127	0.131	0.130	0.133	0.134	0.132	0.135	0.135	0.134
0.134	0.073	0.132	0.135	0.134	0.143	0.135	0.145	0.145	0.145	0.148	0.148	0.147	0.146	0.145	0.148
0.138	0.074	0.136	0.136	0.135	0.144	0.139	0.146	0.146	0.146	0.148	0.150	0.147	0.147	0.147	0.149
0.137	0.075	0.135	0.135	0.134	0.143	0.138	0.145	0.145	0.145	0.147	0.149	0.146	0.146	0.146	0.148
0.139	0.074	0.137	0.138	0.137	0.143	0.138	0.147	0.144	0.147	0.149	0.150	0.147	0.150	0.147	0.147
0.139	0.074	0.137	0.136	0.135	0.145	0.139	0.147	0.148	0.147	0.149	0.151	0.148	0.149	0.150	0.151
0.136	0.074	0.133	0.136	0.136	0.145	0.137	0.146	0.146	0.146	0.148	0.149	0.148	0.146	0.146	0.149
0.141	0.087	0.139	0.138	0.140	0.150	0.139	0.151	0.152	0.153	0.155	0.156	0.150	0.151	0.151	0.153
0.142	0.078	0.141	0.141	0.139	0.148	0.143	0.150	0.149	0.149	0.152	0.155	0.148	0.151	0.152	0.153
0.138	0.085	0.135	0.138	0.138	0.150	0.138	0.148	0.148	0.151	0.152	0.152	0.148	0.148	0.149	0.150
0.137	0.087	0.135	0.137	0.138	0.149	0.137	0.148	0.149	0.152	0.152	0.153	0.147	0.148	0.148	0.151
0.132	0.055	0.129	0.133	0.132	0.142	0.131	0.141	0.142	0.142	0.143	0.146	0.142	0.143	0.141	0.145
0.134	0.058	0.131	0.133	0.132	0.143	0.129	0.141	0.141	0.142	0.144	0.149	0.143	0.141	0.142	0.145
0.134	0.062	0.131	0.135	0.134	0.147	0.133	0.142	0.141	0.143	0.145	0.148	0.143	0.143	0.141	0.146
0.220	0.219	0.217	0.219	0.219	0.224	0.220	0.217	0.218	0.218	0.219	0.220	0.220	0.218	0.217	0.217
0.218	0.219	0.219	0.220	0.220	0.226	0.223	0.220	0.220	0.221	0.221	0.223	0.222	0.222	0.221	0.221
0.223	0.232	0.222	0.227	0.224	0.232	0.226	0.229	0.231	0.229	0.227	0.229	0.230	0.231	0.231	0.229
0.224	0.224	0.222	0.224	0.221	0.224	0.222	0.223	0.224	0.223	0.225	0.226	0.222	0.222	0.225	0.224
0.135	0.084	0.135	0.139	0.138	0.150	0.138	0.147	0.147	0.150	0.151	0.152	0.146	0.148	0.147	0.148
0.135	0.068	0.130	0.134	0.131	0.143	0.135	0.143	0.144	0.144	0.146	0.149	0.144	0.146	0.146	0.145
0.135	0.069	0.136	0.136	0.137	0.145	0.136	0.145	0.143	0.146	0.147	0.151	0.146	0.146	0.145	0.148
0.126	0.152	0.122	0.129	0.126	0.134	0.129	0.037	0.051	0.042	0.039	0.042	0.056	0.067	0.062	0.037
0.224	0.224	0.222	0.223	0.221	0.223	0.223	0.222	0.222	0.223	0.224	0.225	0.221	0.221	0.223	0.223
0.223	0.223	0.221	0.222	0.220	0.223	0.222	0.223	0.223	0.224	0.224	0.225	0.222	0.223	0.223	0.223
0.279	0.276	0.282	0.282	0.282	0.286	0.282	0.287	0.287	0.286	0.286	0.289	0.288	0.287	0.286	0.287

55 56 57 58 59 60 61 62 63 64 65 66 67 68 69 70

0.129															
0.144	0.145														
0.150	0.151	0.084													
0.145	0.146	0.078	0.085												
0.145	0.147	0.078	0.085	0.008											
0.231	0.232	0.228	0.230	0.231	0.230										
0.145	0.147	0.080	0.086	0.010	0.008	0.228									
0.145	0.145	0.079	0.084	0.010	0.009	0.229	0.009								
0.147	0.150	0.084	0.089	0.024	0.022	0.231	0.022	0.022							
0.145	0.147	0.080	0.085	0.011	0.009	0.230	0.009	0.006	0.023						
0.230	0.238	0.226	0.235	0.231	0.229	0.162	0.229	0.229	0.230	0.228					
0.229	0.238	0.227	0.235	0.231	0.230	0.161	0.229	0.229	0.230	0.228	0.003				
0.228	0.237	0.227	0.236	0.231	0.230	0.161	0.229	0.229	0.230	0.228	0.004	0.002			
0.130	0.029	0.147	0.151	0.147	0.147	0.232	0.147	0.146	0.150	0.148	0.237	0.236	0.236		
0.130	0.029	0.146	0.151	0.146	0.147	0.232	0.147	0.146	0.150	0.147	0.237	0.237	0.236	0.001	
0.230	0.238	0.226	0.233	0.231	0.229	0.162	0.230	0.229	0.230	0.228	0.007	0.007	0.008	0.236	0.236
0.131	0.030	0.146	0.151	0.147	0.147	0.233	0.147	0.146	0.150	0.147	0.238	0.237	0.237	0.002	0.002
0.131	0.030	0.146	0.152	0.147	0.147	0.232	0.147	0.146	0.150	0.147	0.237	0.237	0.236	0.002	0.002
0.130	0.034	0.147	0.151	0.149	0.149	0.233	0.149	0.148	0.152	0.150	0.236	0.235	0.235	0.014	0.014
0.130	0.034	0.148	0.152	0.150	0.150	0.233	0.150	0.149	0.153	0.151	0.238	0.237	0.236	0.014	0.013
0.133	0.039	0.151	0.155	0.150	0.151	0.232	0.152	0.150	0.155	0.152	0.238	0.237	0.236	0.020	0.020
0.135	0.041	0.152	0.154	0.151	0.151	0.232	0.151	0.150	0.155	0.151	0.238	0.237	0.237	0.021	0.022
0.135	0.041	0.152	0.154	0.152	0.152	0.231	0.152	0.151	0.155	0.152	0.238	0.237	0.237	0.022	0.022
0.227	0.237	0.227	0.235	0.230	0.229	0.161	0.228	0.229	0.230	0.228	0.019	0.018	0.019	0.237	0.236
0.133	0.039	0.152	0.155	0.150	0.151	0.232	0.152	0.150	0.155	0.152	0.238	0.237	0.236	0.020	0.020
0.133	0.039	0.151	0.155	0.150	0.151	0.231	0.152	0.150	0.155	0.152	0.238	0.237	0.236	0.020	0.020
0.132	0.033	0.149	0.155	0.149	0.149	0.231	0.149	0.148	0.152	0.149	0.237	0.236	0.236	0.013	0.013
0.150	0.150	0.083	0.007	0.082	0.083	0.231	0.084	0.082	0.087	0.083	0.235	0.236	0.236	0.150	0.150
0.132	0.034	0.150	0.153	0.149	0.149	0.233	0.149	0.148	0.153	0.149	0.238	0.237	0.236	0.012	0.012
0.145	0.146	0.024	0.084	0.081	0.079	0.229	0.081	0.080	0.086	0.081	0.227	0.228	0.228	0.146	0.146
0.145	0.146	0.027	0.083	0.081	0.080	0.227	0.081	0.080	0.085	0.081	0.227	0.227	0.227	0.148	0.148
0.145	0.146	0.028	0.084	0.081	0.080	0.228	0.081	0.081	0.085	0.082	0.227	0.228	0.228	0.147	0.147
0.145	0.144	0.080	0.089	0.030	0.029	0.233	0.031	0.031	0.043	0.032	0.231	0.232	0.232	0.144	0.143
0.149	0.147	0.029	0.085	0.082	0.081	0.227	0.082	0.082	0.086	0.083	0.228	0.229	0.229	0.148	0.148
0.146	0.148	0.024	0.083	0.080	0.079	0.228	0.081	0.080	0.086	0.081	0.228	0.229	0.229	0.148	0.147
0.151	0.151	0.086	0.011	0.085	0.085	0.232	0.086	0.085	0.090	0.086	0.234	0.235	0.235	0.153	0.152
0.146	0.149	0.085	0.092	0.035	0.036	0.227	0.038	0.038	0.049	0.038	0.231	0.232	0.232	0.149	0.149
0.149	0.150	0.084	0.012	0.083	0.084	0.228	0.085	0.083	0.086	0.084	0.233	0.233	0.233	0.151	0.150
0.149	0.149	0.085	0.015	0.083	0.084	0.228	0.085	0.083	0.087	0.085	0.234	0.235	0.235	0.150	0.150
0.142	0.146	0.060	0.058	0.063	0.061	0.232	0.062	0.061	0.068	0.063	0.227	0.227	0.227	0.146	0.146
0.142	0.146	0.065	0.064	0.068	0.067	0.234	0.069	0.067	0.073	0.069	0.230	0.231	0.230	0.146	0.146
0.142	0.148	0.064	0.065	0.068	0.067	0.231	0.068	0.067	0.074	0.069	0.229	0.230	0.230	0.150	0.150
0.218	0.225	0.217	0.216	0.215	0.216	0.176	0.216	0.215	0.216	0.216	0.162	0.161	0.161	0.224	0.224
0.222	0.223	0.215	0.220	0.221	0.220	0.159	0.220	0.219	0.219	0.220	0.146	0.146	0.146	0.222	0.222
0.228	0.229	0.231	0.232	0.227	0.227	0.159	0.225	0.225	0.227	0.226	0.146	0.144	0.144	0.227	0.228
0.222	0.225	0.220	0.223	0.221	0.219	0.179	0.220	0.222	0.220	0.222	0.168	0.166	0.167	0.225	0.225
0.146	0.151	0.085	0.033	0.085	0.085	0.232	0.087	0.086	0.088	0.087	0.230	0.230	0.231	0.152	0.152
0.143	0.143	0.068	0.079	0.074	0.072	0.233	0.073	0.071	0.079	0.074	0.226	0.227	0.227	0.144	0.145
0.145	0.146	0.077	0.086	0.081	0.080	0.229	0.080	0.080	0.085	0.081	0.227	0.228	0.228	0.147	0.147
0.058	0.134	0.149	0.154	0.152	0.151	0.229	0.151	0.152	0.154	0.152	0.231	0.230	0.230	0.134	0.134
0.220	0.224	0.222	0.223	0.222	0.220	0.180	0.222	0.222	0.221	0.223	0.168	0.167	0.167	0.225	0.225
0.220	0.223	0.221	0.223	0.221	0.220	0.180	0.221	0.222	0.219	0.222	0.168	0.167	0.168	0.224	0.224
0.287	0.286	0.281	0.277	0.279	0.279	0.287	0.279	0.278	0.281	0.279	0.286	0.286	0.286	0.285	0.285

71	72	73	74	75	76	77	78	79	80	81	82	83	84	85	86

0.237															
0.236	0.002														
0.235	0.014	0.014													
0.237	0.014	0.014	0.004												
0.237	0.021	0.021	0.025	0.026											
0.237	0.022	0.022	0.029	0.028	0.027										
0.237	0.023	0.023	0.029	0.029	0.028	0.001									
0.021	0.237	0.237	0.235	0.236	0.237	0.237	0.237								
0.237	0.021	0.021	0.025	0.025	0.002	0.027	0.028	0.236							
0.237	0.021	0.021	0.025	0.026	0.001	0.027	0.028	0.236	0.002						
0.236	0.013	0.013	0.019	0.019	0.024	0.026	0.027	0.235	0.024	0.024					
0.233	0.150	0.151	0.150	0.152	0.155	0.154	0.154	0.235	0.155	0.154	0.154				
0.237	0.013	0.013	0.019	0.019	0.023	0.025	0.025	0.237	0.023	0.023	0.017	0.153			
0.227	0.146	0.146	0.147	0.148	0.151	0.151	0.151	0.229	0.151	0.150	0.149	0.083	0.149		
0.226	0.148	0.149	0.149	0.150	0.154	0.154	0.154	0.228	0.154	0.153	0.150	0.083	0.150	0.028	
0.227	0.147	0.148	0.148	0.149	0.153	0.153	0.153	0.228	0.153	0.152	0.149	0.084	0.149	0.027	0.001
0.231	0.144	0.144	0.146	0.147	0.149	0.149	0.149	0.231	0.149	0.149	0.146	0.087	0.146	0.082	0.082
0.228	0.148	0.148	0.148	0.149	0.153	0.154	0.154	0.230	0.154	0.153	0.150	0.085	0.150	0.029	0.013
0.227	0.147	0.148	0.149	0.149	0.153	0.154	0.154	0.230	0.153	0.152	0.151	0.082	0.151	0.005	0.026
0.232	0.152	0.153	0.152	0.153	0.156	0.156	0.156	0.236	0.156	0.156	0.156	0.012	0.154	0.085	0.086
0.232	0.149	0.149	0.151	0.153	0.153	0.155	0.155	0.230	0.154	0.153	0.152	0.089	0.152	0.086	0.087
0.231	0.150	0.151	0.150	0.152	0.155	0.154	0.154	0.233	0.155	0.155	0.154	0.011	0.153	0.083	0.083
0.232	0.150	0.150	0.149	0.151	0.154	0.153	0.153	0.234	0.154	0.154	0.153	0.014	0.152	0.084	0.084
0.226	0.146	0.147	0.147	0.149	0.151	0.152	0.152	0.229	0.151	0.150	0.149	0.057	0.149	0.061	0.061
0.229	0.146	0.146	0.148	0.149	0.151	0.153	0.153	0.231	0.151	0.150	0.149	0.063	0.149	0.067	0.066
0.228	0.150	0.150	0.151	0.152	0.153	0.156	0.156	0.230	0.154	0.153	0.152	0.064	0.152	0.066	0.066
0.161	0.224	0.224	0.224	0.223	0.227	0.228	0.227	0.163	0.227	0.227	0.225	0.215	0.226	0.221	0.215
0.146	0.223	0.222	0.225	0.226	0.224	0.223	0.223	0.148	0.224	0.223	0.221	0.221	0.223	0.218	0.218
0.144	0.228	0.228	0.228	0.229	0.229	0.227	0.227	0.148	0.229	0.229	0.227	0.232	0.229	0.232	0.230
0.166	0.226	0.225	0.226	0.227	0.227	0.228	0.227	0.168	0.227	0.227	0.227	0.222	0.227	0.221	0.220
0.228	0.151	0.152	0.151	0.153	0.156	0.155	0.155	0.231	0.156	0.155	0.155	0.031	0.154	0.085	0.085
0.227	0.145	0.144	0.146	0.147	0.147	0.150	0.150	0.229	0.146	0.146	0.148	0.078	0.148	0.068	0.070
0.227	0.147	0.147	0.150	0.150	0.152	0.151	0.152	0.228	0.152	0.151	0.151	0.085	0.150	0.076	0.076
0.231	0.135	0.134	0.136	0.135	0.138	0.138	0.137	0.230	0.137	0.138	0.137	0.155	0.137	0.150	0.152
0.167	0.225	0.225	0.226	0.227	0.227	0.228	0.227	0.168	0.227	0.226	0.227	0.221	0.227	0.222	0.221
0.167	0.225	0.225	0.225	0.226	0.226	0.226	0.226	0.169	0.226	0.226	0.227	0.222	0.226	0.222	0.221
0.285	0.286	0.285	0.287	0.287	0.288	0.286	0.286	0.287	0.288	0.288	0.284	0.277	0.286	0.280	0.281

87 88 89 90 91 92 93 94 95 96 97 98 99 100 101 102

0.082															
0.014	0.083														
0.027	0.082	0.028													
0.087	0.091	0.087	0.085												
0.087	0.046	0.087	0.085	0.094											
0.084	0.088	0.084	0.082	0.017	0.092										
0.085	0.089	0.085	0.083	0.020	0.091	0.004									
0.062	0.066	0.061	0.060	0.060	0.071	0.057	0.059								
0.067	0.070	0.067	0.066	0.066	0.074	0.065	0.067	0.019							
0.067	0.072	0.067	0.065	0.067	0.074	0.063	0.065	0.023	0.031						
0.216	0.217	0.217	0.220	0.215	0.217	0.214	0.213	0.212	0.213	0.212					
0.218	0.222	0.220	0.219	0.221	0.222	0.218	0.218	0.218	0.219	0.218	0.158				
0.230	0.231	0.233	0.231	0.232	0.229	0.230	0.230	0.225	0.230	0.228	0.162	0.132			
0.220	0.222	0.223	0.221	0.224	0.220	0.222	0.222	0.221	0.223	0.221	0.158	0.163	0.161		
0.086	0.089	0.085	0.084	0.036	0.093	0.033	0.036	0.060	0.065	0.065	0.214	0.220	0.230	0.221	
0.071	0.080	0.070	0.069	0.080	0.082	0.077	0.079	0.052	0.057	0.057	0.218	0.217	0.226	0.226	0.080
0.077	0.082	0.077	0.075	0.087	0.086	0.084	0.085	0.064	0.066	0.067	0.219	0.219	0.223	0.229	0.085
0.151	0.150	0.153	0.152	0.157	0.153	0.154	0.154	0.146	0.147	0.148	0.222	0.224	0.233	0.226	0.152
0.222	0.222	0.224	0.221	0.223	0.221	0.221	0.222	0.222	0.225	0.222	0.158	0.162	0.161	0.015	0.221
0.221	0.220	0.223	0.222	0.224	0.220	0.223	0.223	0.222	0.223	0.222	0.159	0.162	0.161	0.016	0.221
0.281	0.277	0.282	0.281	0.278	0.278	0.277	0.277	0.279	0.280	0.278	0.285	0.285	0.283	0.286	0.279

103 104 105 106 107 108

0.068				
0.149	0.153			
0.226	0.228	0.224		
0.226	0.229	0.224	0.012	
0.282	0.280	0.290	0.285	0.286

Table. Estimates of Evolutionary Divergence between Sequences

The number of base differences per site from between sequences are shown. The analysis involved 108 nucleotide sequences. Codon positions included were 1st+2nd+3rd+Noncoding. All positions containing gaps and missing data were eliminated. There were a total of 5079 positions in the final dataset. Evolutionary analyses were conducted in MEGA7 [1].

1. Kumar S., Stecher G., and Tamura K. (2016). MEGA7: Molecular Evolutionary Genetics Analysis version 7.0 for bigger datasets.Molecular Biology and Evolution 33:1870-1874.

Disclaimer: Although utmost care has been taken to ensure the correctness of the caption, the caption text is provided "as is" without any warranty of any kind. Authors advise the user to carefully check the caption prior to its use for any purpose and report any errors or problems to the authors immediately (www.megasoftware.net). In no event shall the authors and their employers be liable for any damages, including but not limited to special, consequential, or other damages. Authors specifically disclaim all other warranties expressed or implied, including but not limited to the determination of suitability of this caption text for a specific purpose, use, or application.
