# Supplementary material for: Simultaneous Occurrence of Field Epidemics of Rabbit Hemorrhagic Disease (RHD) in Poland Due to the Co-Presence of Lagovirus europaeus GI.1 (RHDV)/GI.1a (RHDVa) and GI.2 (RHDV2) Genotypes
Source: Viruses. 2025 Sep 26;17(10):1305. doi: 10.3390/v17101305 (PMC12568209; doi:10.3390/v17101305)
Supplement: Supplementary file 1 [file viruses-17-01305-s001.zip › Table S1 List of lagovirus sequences.pdf]

List of lagovirus sequences used for NSP (1-108) and VP60 (1-111) phylogenesis and to root the tree.

1 WAE_2022_(OR488784)_PL
2 ZWO_2021_(OQ605827)_PL
3 KOB_2020_(OQ605828)_PL
4 LIB_2020_(OQ605829)_PL
5 NRU_2020_(OQ605830)_PL
6 PD_1989_(KP144789)_PL
7 MAL_1994_(KU882093)_RHDV_PL
8 BLA_1994_(KP144792)_PL
9 OPO_2004_(KU882094)_PL
10 GSK_1998_(KU882092)_PL
11 ZD0_2000_(KU882095)_PL
12 GRZ_2004_(KP144791)_PL
13 L14504_2004_(KY679902)_PL
14 W14705_2005_(KY319035)_PL
15 STR_2012_(KF677011)_PL
16 GLE_2013_(KY319032)_PL
17 SKO_2013_(KY319034)_PL
18 RED1_2013_(KY679903)_PL
19 STR2_2013_(KY679904)_PL
20 STR_2014_(KY679905)_PL
21 BIE_2015_(KY319031)_PL
22 F77-3_2015_(MN853658)_PL
23 BBI_2017_(MG602005)_PL
24 RED_2016_(MG602006)_PL
25 VMS_2017_(MG602007)_PL
26 PIN_2018_(MN853660)_PL
27 LIB_2018_(MN853659)_PL
28 WAK_2018_(MN853661)_PL
29 V351_1987_(U54983)_CZ
30 FRG_1989_(M67473)_DE
31 Jena_1993_(EF5585760)_DE
32 Frankfurt5_1996_(EF558573)_DE
33 AST89_1989_(Z49271)_ES
34 RHDV-SD_1989_(Z29514)_FR
35 95-10_1995_(MT628287)_FR
36 00-21_2000_(MH190418)_FR
37 09-02_2009_(MT628289)_FR
38 09-03_2009_(MT628290)_FR
39 96VLT000113_1995_(MT819374)_S
40 12VLT000099_2012_(MT819377)_S
41 BS89_1989_(X87607)_IT
42 CB137_1995_(JX886002)_PT
43 CB156_1997_(JF438967)_PT
44 CB194_2006_(JX886001)_PT
45 Woodcroft_2005_(KT006741)_AUS
46 Triptis_1996_(EF558583)_DE
47 Erfurt_1996_(EF558581)_DE
48 Rossi_2002_(EF558584)_DE
49 P175_1999_(KY622129)_PT
50 RHDV-Hokkaido_2002_(AB300693)_JPN

51	WHNRH_2005_(DQ280493)_CN
52	RHDV_2014_(MK895974)_CN
53	WIN-AH-2011-OTH-026_(KY235676)_CAN
54	Iowa_2000_(AF258618)_USA
55	IN-05_2005_(EU003578)_USA
56	13-165_2013_(MN737112)_FR
57	16-35OOd_2016_(MN738377)_FR
58	NL-2016_(MN061492)_NL
59	N11_2011_(KM87868)_ES
60	Zar11-11_2010_(KP129398)_ES
61	CBAnd1_2012_(KP090976)_ES
62	Seg08-12_2012_(KP129396)_ES
63	Rij06-12_2012_(KP129395)_ES
64	16PLM1_2016_(MF407653)_ES
65	CBVal16_2012_(KM979445)_PT
66	Algarve1_2013_(KF442961)_PT
67	7-13_Barrancos_2013_(KF442963)_PT
68	10A-13_Barrancos_2013_(KF442964)_PT
69	CBAlgarve14-1_2014_(KM115714)_PT
70	CBAlgarve14-3_2014_(KM115715)_PT
71	CBEstremoz_14-1_2014_(KM115681)_PT
72	CBMert_14-1_2014_(KM115712)_PT
73	CBMert14-2_2014_(KM115713)_PT
74	SOS089_2014_(MG763936)_PT
75	SOS158_2015_(MG763947)_PT
76	PSM2_2016_(MF407654)_PT
77	CBPico17-1_2017_(MF407651)_PT
78	CBPico-17-2_2017_(MF407652)_PT
79	CBMad17-1_2017_(MF407655)_PT
80	CBMad17-2_2017_(MF407656)_PT
81	CBMad17-3_2017_(MF407657)_PT
82	RHDV/GER-NW/D51-1.L00911_2014_(LR899189)_DE
83	EI327.L03607/2016_(LR899157)_DE
84	BLMT-1_2015_(KT280060)_AUS
85	AZ1_2020_(MT506237)_USA
86	NY1_2020_(MT506236)_USA
87	NY2_2020_(MT506235)_USA
88	WIN-AH-2016-OTH-0018_(KY235675)_CAN
89	WIN-AH-2019-OTH-0032_(MT900574)_CAN
90	Senasica20_2020_(OM973948)_MX
91	RHDV2-S25_2019_(MW789242)_GH
92	Touza_1_2019_(MZ913394)_TN
93	Ibaraki-1_2019_(LC749423)_JPN
94	Chiba-1_2020_(LC749425)_JPN
95	06-11_2006_(MN737115)_FR
96	08-133_2008_(MN746289)_FR
97	09-48_2009_(MN737116)_FR
98	MRCV_2001_(GQ166866)_USA
99	RCV-A1_MIC-07_2007_(EU871528)_AUS
100	Australia_1_2007_(KX357690)_AUS
101	AUS/NSW/ANN-1/2014/04_(KY628306)_AUS
102	Otago/NZ-37/2018_(OM372665)_NZ
103	10-28_2010_(MN737113)_FR

104	10-32_2010_(MN737114)_FR
105	K5_08Q712_2008_(MF598301)_AUS
106	ACT/AIN-5_2017_(MW460019)_AUS
107	NSW/CAR-3/2016_(MF598302)_AUS
108	EBHSV-GD_(Z69620)_FR
109	Ca11_2011_(KC345614)_IT
110	NanBu_2011_(JQ815391)_CN
111	RCV_1996_(X96868)_IT
